# Advanced single-cell and spatial analysis with high-multiplex characterization of circulating tumor cells and tumor tissue in prostate cancer: Unveiling resistance mechanisms with the CoDuCo in situ assay

**DOI:** 10.1186/s40364-024-00680-z

**Published:** 2024-11-16

**Authors:** Lilli Bonstingl, Margret Zinnegger, Katja Sallinger, Karin Pankratz, Christin-Therese Müller, Elisabeth Pritz, Corinna Odar, Christina Skofler, Christine Ulz, Lisa Oberauner-Wappis, Anatol Borrás-Cherrier, Višnja Somođi, Ellen Heitzer, Thomas Kroneis, Thomas Bauernhofer, Amin El-Heliebi

**Affiliations:** 1 Division of Cell Biology, Histology and Embryology, Gottfried Schatz Research Center, Medical University of Graz, 8010 Graz, Austria; 2 Center for Biomarker Research in Medicine (CBmed), 8010 Graz, Austria; 3 European Liquid Biopsy Society (ELBS), 20246 Hamburg, Germany; 4 Diagnostic and Research Center for Molecular BioMedicine, Diagnostic & Research Institute of Pathology, Medical University of Graz, 8010 Graz, Austria; 5 Division of Oncology, Department of Internal Medicine, Medical University of Graz, 8010 Graz, Austria; 6 Diagnostic and Research Center for Molecular BioMedicine, Institute of Human Genetics, Medical University of Graz, 8010 Graz, Austria; 7 Christian Doppler Laboratory for Liquid Biopsies for Early Detection of Cancer, Medical University of Graz, 8010 Graz, Austria; 8 University Comprehensive Cancer Center (CCC) Graz, 8010 Graz, Austria

**Keywords:** Circulating tumor cells (CTCs), Metastatic prostate cancer, Multiplex padlock probe in situ hybridization, Single-cell gene expression, Liquid biopsy, Spatial transcriptomics, Neuroendocrine transdifferentiation, Resistance monitoring, Image analysis

## Abstract

**Background:**

Metastatic prostate cancer is a highly heterogeneous and dynamic disease and practicable tools for patient stratification and resistance monitoring are urgently needed. Liquid biopsy analysis of circulating tumor cells (CTCs) and circulating tumor DNA are promising, however, comprehensive testing is essential due to diverse mechanisms of resistance. Previously, we demonstrated the utility of mRNA-based in situ padlock probe hybridization for characterizing CTCs.

**Methods:**

We have developed a novel combinatorial dual-color (CoDuCo) assay for in situ mRNA detection, with enhanced multiplexing capacity, enabling the simultaneous analysis of up to 15 distinct markers. This approach was applied to CTCs, corresponding tumor tissue, cancer cell lines, and peripheral blood mononuclear cells for single-cell and spatial gene expression analysis. Using supervised machine learning, we trained a random forest classifier to identify CTCs. Image analysis and visualization of results was performed using open-source Python libraries, CellProfiler, and TissUUmaps.

**Results:**

Our study presents data from multiple prostate cancer patients, demonstrating the CoDuCo assay’s ability to visualize diverse resistance mechanisms, such as neuroendocrine differentiation markers (SYP, CHGA, NCAM1) and AR-V7 expression. In addition, druggable targets and predictive markers (PSMA, DLL3, SLFN11) were detected in CTCs and formalin-fixed, paraffin-embedded tissue. The machine learning-based CTC classification achieved high performance, with a recall of 0.76 and a specificity of 0.99.

**Conclusions:**

The combination of high multiplex capacity and microscopy-based single-cell analysis is a unique and powerful feature of the CoDuCo in situ assay. This synergy enables the simultaneous identification and characterization of CTCs with epithelial, epithelial-mesenchymal, and neuroendocrine phenotypes, the detection of CTC clusters, the visualization of CTC heterogeneity, as well as the spatial investigation of tumor tissue. This assay holds significant potential as a tool for monitoring dynamic molecular changes associated with drug response and resistance in prostate cancer.

## Background

Prostate cancer (PC) is the third most frequently diagnosed solid cancer worldwide, with an estimated 1.4 million new cases reported in 2020 [1, 2]. The risk for developing invasive PC increases with age and is highest for men older than 69 years [3, 4]. With a world population that is growing and aging persistently, further increase of PC incidence is to be expected. Indeed, projections based on demographic changes and rising life expectancy suggest that annual new PC cases will increase to 2.9 million by 2040 [5].

Despite continuous improvements in treatment options for PC, the therapy of advanced PC is challenging, since the selective pressure created by new treatments also promotes the emergence of resistance mechanisms [6–8]. Dysregulation of the androgen receptor (AR) pathway plays a key role in the development and progression of PC. Consequently, the most common treatment for advanced disease is androgen deprivation therapy (ADT). Although promising results are obtained, patients develop a high rate of resistance to ADT, hence classified as castration resistant prostate cancer (CRPC) [7]. Novel AR-targeted treatments such as enzalutamide and abiraterone in combination with ADT represent effective therapy options for hormone-sensitive PC and CPRC patients [9]. However, almost all patients acquire secondary resistance to novel AR-targeted treatments, leading to the progression of the fatal disease [9].

Resistance is frequently driven by aberrations of the AR signaling pathway, including *AR* gene amplification, mutations, and the expression of AR splice variants, particularly the splice variant AR-V7 [10–12]. Additionally, AR-independent mechanisms like neuroendocrine transdifferentiation are on the rise as well [8].

To date, there are no coherent biomarker-based recommendations for optimal individualized treatment combinations and sequences of therapy lines, and clinically practicable tools for patient stratification and monitoring of drug resistance are unavailable [13, 14]. There is an urgent need to investigate a multitude of resistance mechanisms, which could be exploited as predictive biomarkers. For example, AR-V7-positive patients are more likely to benefit from taxane-based chemotherapy than AR-targeted drugs, while platinum-based chemotherapy may be indicated in patients with neuroendocrine PC [15, 16]. Moreover, other treatments, such as drugs targeting PSMA or DLL3-expressing tumor cells, are increasingly available or under investigation [17, 18].

In recent years, the concept of liquid biopsy gained tremendous attention as a minimally invasive way to monitor disease state using different analytes such as circulating tumor cells (CTCs) and circulating tumor DNA (ctDNA) [19–21]. However, a major challenge in liquid biopsies lies in simultaneously investigating a broad spectrum of resistance mechanisms and predictive biomarkers in PC. Relevant alterations range from genetic aberrations (e.g. *AR*-gain or mutations) [22] to transcriptional changes such as alternative splicing (e.g. AR-V7) [23–25], expression of PSMA and SLFN11 [26, 27], or upregulation of neuroendocrine markers (e.g. SYP, CHGA, NCAM1, and DLL3) in cancers cells [8]. The current status of CTC biomarker detection commonly relies on panels of markers, typically assessed through antibody staining or PCR techniques. For example, PSMA expression in CTCs can be analyzed at both the mRNA and protein levels [28–32]. Neuroendocrine differentiation in CTCs can be detected based on morphological features [33], through qPCR-based assays [34, 35], or via in situ approaches [36]. A significant limitation is the investigating of multiple resistance mechanisms simultaneously, particularly when using antibody staining with a limited number of fluorescence channels. To gain a comprehensive overview of resistance mechanisms and druggable targets, a multiplex liquid biopsy assays is essential.. While ctDNA analysis excels in uncovering genetic alterations, transcriptional regulation analysis of single gene loci by ctDNA nucleosome patterns remains challenging [37]. Previously, we have demonstrated that resistance mechanisms in CTCs can be characterized by mRNA-based in situ padlock probe (PLP) hybridization, using an assay that provided broad expression data of AR, AR-V7, and PSA in CTCs of PC patients [38]. The main technical challenges of in situ PLP-based CTC analysis were time-intensive manual evaluation of in situ data and restricted multiplexability of the existing technique (e.g. limitation to detect 3 transcripts on a 4-channel fluorescence microscope).

Building on this, we set out to provide an advanced multiplex mRNA-based in situ assay that reveals additional predictive biomarkers in CTCs. Through machine learning algorithms, the time-intensive image evaluation of CTCs is streamlined, thereby overcoming the aforementioned limitations.

Here, we present our achievements in developing a multiplex mRNA-based CTC assay examining multiple biomarkers with significant predictive power, including PSMA, PSA, AR, AR-V7, and neuroendocrine markers SYP, CHGA, and NCAM1. Our innovative approach introduces a novel combinatorial dual-color (CoDuCo) in situ hybridization assay with increased multiplex capacity of up to 15 targets, semi-automated image analysis, and machine learning-assisted CTC classification with a turnaround time of 3–4 days.

## Methods

### Patient Sampling and Ethics

The study enrolled patients with advanced metastatic PC at the Division of Oncology, Department of Internal Medicine, Medical University of Graz (Austria), following the principles of the World Medical Association Declaration of Helsinki. The study was approved by the ethics committee (EK 31–353 ex 18/19) and written informed consent was obtained from all patients and healthy controls. To avoid contamination by epithelial cells, one extra blood tube with 2.5 ml of blood was collected for non-cell-based analyses before collecting the blood samples for cell-based analyses. For isolation of peripheral blood mononuclear cells (PBMCs) from healthy controls, blood samples were collected in VACUETTE blood collection tubes K3E K3EDTA (9 mL blood) (Greiner Bio-One, Kremsmünster, Austria). For CTC enrichment, blood samples were collected in 8.5 ml BD Vacutainer ACD-A tubes (BD Switzerland Sarl, Eysins, Switzerland). All blood samples were collected following the CEN/TS 17390–3 standards to ensure defined pre-analytical parameters as described previously [39].

### Cell line and PBMC sample preparation

PC cell lines VCaP (kindly provided by Martina Auer, Medical University of Graz, Graz, Austria) and PC-3 (American Type Culture Collection (ATCC), Manassas, VA, USA) were cultured as described in detail by Hofmann, Kroneis and El-Heliebi [40]. The lung cancer cell line NCI-H1299 (kindly provided by Eva Obermayr, Medical University of Vienna, Vienna, Austria) was cultured in RPMI (Roswell Park Memorial Institute) 1640 Medium (Thermo Fisher Scientific, Waltham, MA, USA) with 10% FBS and 1% Penicillin/Streptomycin at 37°C and 5% CO_2_. All cell lines were harvested as published previously [40]. For in situ assay validation experiments, the PBMC fraction of healthy donors’ blood samples in VACUETTE blood collection tubes K3E K3EDTA was isolated by density gradient centrifugation as described previously [38]. PBMCs, VCaP, PC-3, and NCI-H129 cells were fixed in 3.7% formaldehyde (Sigma-Aldrich, St Louis, MO, USA, catalog number F1635) in PBS (Thermo Fisher Scientific, catalog number 10010015) for 5 min, resuspended in 1 × PBS, and 1 × 10^5^ cells were transferred to SuperFrost Plus microscope slides (Thermo Fisher Scientific, catalog number J1800AMNT) by cytocentrifugation using a Hettich Universal 32 benchtop centrifuge. Slides were dried over night at room temperature and stored at -80°C.

### CTC enrichment and sample preparation for in situ analysis

CTCs were enriched from 7.5 ml of blood samples collected in ACD-A tubes using the Cytogen Smart Biopsy Cell Isolator (Cytogen Inc., Seoul, Korea) following the manufacturer’s protocol. In short, the double negative selection workflow involves incubation with a leukocyte and erythrocyte depletion cocktail and subsequent density gradient centrifugation. Samples are then forwarded to the automated Smart Biopsy Cell Isolator, which performs a size-based filtration using a high-density microporous (HDM) chip, retrieval of the enriched cell fraction from the HDM chip, and automated transfer to a reaction tube [41]. The cells were fixed with 2% formaldehyde for 5 min and centrifuged on Micro Slide Glass Frontier FRC-01 (Matsunami Glass Industry Ltd, Osaka, Japan) using a Shandon Cytospin 2. The cells were washed 3 times with PBS, dried over night at room temperature and stored at -80°C. This procedure was applied to patient blood samples, healthy controls’ blood samples, and healthy controls’ blood samples spiked with 500 VCaP and 500 PC-3 cells.

### CoDuCo in situ PLP hybridization

In situ PLP hybridization with a novel CoDuCo staining approach was used to visualize transcripts in cells. In situ PLP hybridization starts with targeted reverse transcription. Then, PLPs are hybridized to the cDNA and ligated to form closed circular molecules. The PLP sequence is amplified by rolling circle amplification and finally, bridge probes and fluorescently labelled readout detection probes are hybridized to the resulting rolling circle products (RCPs) [40, 42, 43].

#### Selection of genes

CoDuCo in situ hybridization was used to visualize hematopoietic transcripts *PTPRC* (protein tyrosine phosphatase receptor type C (CD45), GenBank accession number NM_002838.5), *ITGAM* (integrin subunit alpha M (CD11B), NM_000632.4), *FCGR3A* (Fc gamma receptor IIIa (CD16), NM_001127596.2), *FCGR3B* (Fc gamma receptor IIIb (CD16), NM_001271035.2), *CD4* (CD4 molecule, NM_001195017.3), and *ITGB2* (integrin subunit beta 2 (CD18), NM_000211.5); epithelial transcripts *EPCAM* (epithelial cell adhesion molecule, NM_002354.3), *KRT8* (keratin 8, NM_001256293.2), *KRT18* (keratin 18, NM_000224.3), and *KRT19* (keratin 19, NM_002276.5); prostate-specific transcripts *KLK3* (kallikrein related peptidase 3 (prostate specific antigen PSA), NM_001030047), *FOLH1* (folate hydrolase 1 (prostate specific membrane antigen PSMA), NM_004476.3), *AR-FL* (androgen receptor full length, NM_000044.3), and *AR-V7* (androgen receptor splice variant 7, FJ235916.1); neuroendocrine transcripts *SYP* (synaptophysin, NM_003179.3), *CHGA* (chromogranin A, NM_001275.4), *NCAM1* (neural cell adhesion molecule 1, NM_001242607.2), and *DLL3* (delta like canonical Notch ligand 3, NM_203486.3). In addition, *VIM* (vimentin, NM_003380.5) and *SLFN11* (schlafen family member 11, NM_001376010.1) were visualized.

#### Probe design

Reverse transcription primers with a length of 15–25 nucleotides were designed using CLC Main Workbench software (CLC Bio workbench version 7.6, QIAGEN, Hilden, Germany) based on the guidelines published by Weibrecht et al. [44]. Primer binding sites close to the PLP binding sites were preferred and an overlap of up to 6 nucleotides was allowed. Up to 6 locked nucleic acids (LNA)-modified nucleotides were included in selected primers to increase binding strength. No overlap of LNA-modified nucleotides with the PLP-binding site was allowed.

PLPs consist of 3’ and 5’ target-binding arms linked by a central backbone. PLPs were designed using a Python software package developed by the Mats Nilsson Lab, Stockholm University (https://github.com/Moldia/multi_padlock_design), with an arm length of 15 nucleotides and melting temperature between 65°C and 75°C [43]. Some PLP binding sites were determined manually, using CLC Main Workbench software based on the guidelines published by Weibrecht et al. [44]. Manually designed PLPs had 15–19 nucleotide long target-binding arms, covering a binding site of 30–38 nucleotides in total. The PLP backbones contain a 17–20 nucleotide ID sequence unique for each gene. To increase sensitivity of the assay, each gene was targeted by up to 44 reverse transcription primers and up to 20 PLPs.

Bridge probes were used for indirect hybridization of 5’ fluorescently labelled readout detection probes to the RCP. They consist of the 17 nucleotide ID sequence, which binds to the RCP, a 2-nucleotide linker, and the reverse complementary 20 nucleotide sequence of the respective readout detection probes.

Oligonucleotides were ordered from IDT (Integrated DNA Technologies, Coralville, IA, USA) and stored at -20°C as 100 µM stocks or 10 µM dilutions in nuclease-free water (Thermo Fisher Scientific, catalog number AM9930) or IDTE buffer pH 8 (Integrated DNA Technologies). PLPs were ordered 5’ phosphorylated.

#### Buffers for in situ hybridization

Diethyl pyrocarbonate (DEPC, Sigma-Aldrich, catalog number D5758) was used to remove RNase activity in ultrapure water (H_2_O). DEPC-H_2_O (1 ml DEPC in 1 l H_2_O) was incubated overnight at room temperature and then autoclaved to deactivate DEPC. DEPC-H_2_O was used to prepare DEPC-PBS (phosphate buffered saline) and DEPC-PBS-Tween (0.05% Tween-20, Sigma-Aldrich, catalog number 822184).

#### In situ hybridization

Prefixed slides were thawed for 3 min, fixed again with 3.7% formaldehyde in 1 × DEPC-PBS for 15 min, and washed with 1 × DEPC-PBS-Tween for 2 min. Slides were then dehydrated through ascending ethanol series (70%, 85%, and 100% ethanol in DEPC-H_2_O for 2 min each). 50 µl SecureSeal hybridization chambers (Thermo Fisher Scientific, catalog number S24732) were mounted on completely air-dried slides to cover the cytospinned cells. Cells were rehydrated with 1 × DEPC-PBS-Tween for 5 min, permeabilized with 0.1 M HCl (hydrochloric acid, Merck Chemicals and Life Sciences, Vienna, Austria, catalog number 1.09970.0001) in DEPC-H_2_O for 5 min and washed twice with 1 × DEPC-PBS-Tween for 5 min each.

The reaction mix for reverse transcription (RT) contained 40 U/μl TranscriptMe Reverse Transcriptase (DNA-Gdansk, Gdansk, Poland, catalog number RT32-010), 2 U/μl RiboLock RNase inhibitor (Thermo Fisher Scientific, catalog number EO0381), 0.5 mM dNTPs (Sigma-Aldrich, catalog number D7295), 0.1 μM of each reverse transcription primer and 0.4 μg/μl BSA (Thermo Fisher Scientific, catalog number B14) in RT buffer (DNA-Gdansk). Reverse transcription was carried out at 45°C for 3 h, followed by fixation with 3.7% formaldehyde in 1 × DEPC-PBS for 10 min, and two washing steps with 1 × DEPC-PBS-Tween for 2 min each.

The reaction mix for PLP hybridization and ligation contained 1 U/μl Ampligase (Biozym Biotech Trading, Vienna, Austria, catalog number 111075), 0.8 U/μl RNase H (Thermo Fisher Scientific, catalog number EN0201), 0.4 μg/μl BSA, 0.1 μM of each PLP, 0.05 M KCl (potassium chloride, Sigma-Aldrich, catalog number P9333) and 20% formamide (Sigma-Aldrich, catalog number F9037) in Ampligase buffer. Incubation was started at 37°C for 30 min for RNA digestion, followed by 45 min at 45°C for PLP hybridization and ligation. Samples were washed once with pre-warmed 2 × SSC-Tween (saline sodium citrate buffer, Thermo Fisher Scientific, catalog number 15557044; 0.05% Tween-20) at 37°C for 5 min and twice with 1 × DEPC-PBS-Tween at room temperature for 2 min each.

The reaction mix for rolling circle amplification contained 2 U/μl φ29 Polymerase (Thermo Fisher Scientific, catalog number EP0091), 0.4 μg/μl BSA, 0.25 mM dNTPs, and 5% Glycerol (Carl Roth, Karlsruhe, Germany, catalog number 3783.1) in φ29 Polymerase buffer. Amplification was performed overnight at room temperature, followed by two washes with 1 × DEPC-PBS-Tween for 2 min each.

Bridge probes were hybridized at a final concentration of 0.1 µM in a hybridization buffer of 20% formamide in 2 × SSC at 37°C for 60 min, followed by two washes with 2 × SSC for 2 min each. Readout detection probes were hybridized at a final concentration of 0.1 µM together with 2 µg/ml DAPI (Thermo Fisher Scientific, catalog number D21490) in a hybridization buffer of 20% formamide in 2 × SSC at 37°C for 30 min, followed by two washes with 1 × DEPC-PBS-Tween for 2 min each.

SecureSeal hybridization chambers were removed from the slides and the cytospins were covered with SlowFade Gold Antifade Mountant (Thermo Fisher Scientific, catalog number S36936) and a coverslip for imaging.

To increase signal to noise ratios we implemented a mathematical subtraction of background fluorescence. To do so, we first imaged the slides, then removed in situ signals by formamide stripping, rescanned the remaining background fluorescence and subtracted this background from the originally scanned images. In detail, slides were soaked in 1 × DEPC-PBS to gently remove coverglass and mounting medium, dehydrated through ascending ethanol series (70%, 85%, and 100% ethanol in DEPC-H_2_O for 2 min each) and air-dried. 50 µl SecureSeal hybridization chambers were mounted on the slides. Samples were rehydrated with 1 × DEPC-PBS-Tween for 5 min and incubated three times with prewarmed 100% formamide at 37°C, followed by two washes with 1 × DEPC-PBS-Tween for 2 min each. To enhance the DAPI staining, samples were incubated with 2 µg/ml DAPI in 1 × DEPC-PBS at 37°C for 30 min. Samples were washed twice with 1 × DEPC-PBS-Tween for 2 min each. The SecureSeal hybridization chambers were removed, and the samples were mounted for imaging as described before.

Unless noted otherwise, all steps were performed at room temperature.

#### Tissue preparation

For in situ hybridization of tissue samples, formalin-fixed and paraffin-embedded (FFPE) tissue was used. FFPE sections were preprocessed as previously described [45]. In short, 5 µm tissue sections were baked for 1 h at 60 °C, deparaffinized in Histoclear (Sanova Pharma, Vienna, Austria) and permeabilized in a steamer using a pH6 citrate buffer for 45 min. For in situ hybridization, half enzyme concentration was used (i.e. 20 U/μl TranscriptMe Reverse Transcriptase and 1 U/μl RiboLock RNase inhibitor for reverse transcription, 0.5 U/μl Ampligase and 0.4 U/μl RNase H for PLP hybridization and ligation, and 1 U/μl φ29 Polymerase for rolling circle amplification). After in situ hybridization, we implemented quenching of unspecific background fluorescence using TrueBlack Lipofuscin Autofluorescence Quencher (TLAQ) as described previously [43].

#### Imaging

Slides were imaged using Slideview VS200 digital slide scanners (Evident, Tokio, Japan). The scanners were equipped with external LED light sources Xcite Xylis or Xcite Novem (Excelitas, Mississauga, Canada), Olympus universal-plan extended apochromat 40 × objectives (UPLXAPO40x, 0.95 NA/air; Olympus), and Hamamatsu ORCA-Fusion digital sCMOS cameras (C14440-20UP, 2304 × 2304 (5.3 Megapixels), 16 bit; Hamamatsu City, Japan). DAPI, Cy5, and AF750 were imaged using a Semrock penta filter (AHF-LED-DFC3C5C7-5-SBM; AHF, Tübingen-Pfrondorf, Germany / Semrock / IDEX Health & Science, Oak Harbor, Washington, USA) with excitation wavelengths of 378/52 nm, 635/18 nm, and 735/28 nm, and emission wavelengths of 432/36 nm, 685/42 nm, and 809/81 nm. Atto425, Atto488, Cy3, and TexasRed were imaged using Spectrasplit filters (Kromnigon, Goteborg, Sweden) with excitation wavelengths of 438/24 nm, 509/22 nm, 550/10 nm, and 578/21 nm, and emission wavelengths of 482/25 nm, 544/24 nm, 565/16 nm, and 641/75 nm. Exposure times and lamp intensities were adjusted depending on the used light source and sample type (DAPI 0.8–10 ms with 20–100% lamp intensity and Atto425 50–100 ms, Atto488 50–80 ms, Cy3 30–70 ms, TexasRed 40–80 ms, Cy5 20–200 ms, AF750 100–300 ms with 70–100% lamp intensity). Since in situ signals can be localized at different focal planes in cells and especially in larger cell aggregates such as CTC clusters, we used extended focus imaging with a z-range of 7.41 µm and z-spacing of 0.84 µm (9 z-planes) to depict in-focus in situ signals in a single plane.

#### Image analysis of cell-based samples

Original and background scan images were converted to TIF file format (LZW compression) in reduced resolution by 8 × 8 binning as well as in full resolution. The 8 × 8-binned DAPI image was segmented in CellProfiler version 4 (Broad Institute of MIT and Harvard, Cambridge, MA, USA) [46] to detect regions of interests (ROIs) occupied by nuclei. The coordinates of ROI bounding boxes were exported. In addition, a binary image of each ROI was cropped and saved.

The Python package pyStackReg was used for the registration of original and background images [47]. During pre-alignment, a rigid body transformation matrix was computed for the 8 × 8 binned DAPI images. The matrix was multiplied by 8 for upscaling and applied to register the full resolution background image. ROIs were then cropped from the full resolution images based on the coordinates determined in CellProfiler. For optimal image alignment, the rigid body registration based on the DAPI channel was repeated for each cropped full resolution ROI image.

The cropped full resolution images and the binary ROI images were then subjected to a second CellProfiler pipeline for cell segmentation, in situ signal detection and decoding, and cell classification. First, for all channels except DAPI, the background images were subtracted from the original images to decrease autofluorescence of cytoplasm and other objects such as erythrocytes, thereby increasing the relative intensity of in situ signals [45]. The binary ROI image was used to mask the DAPI image to remove nuclei of neighboring ROIs with overlapping bounding boxes. High and low intensity nuclei were identified by separate instances of the “IdentifyPrimaryObjects” module and were then combined into a single object set. Cell borders were identified by expanding nuclei by 12 pixel (px; 2 µm) or 32 px (5 µm) depending on nucleus area below or above 2800 px (73.6 µm^2^; radius 4.8 µm). In samples that contained cells with particularly large cytoplasmic volume, an additional propagation step, guided by autofluorescence in the Atto488 background scan, was included. In situ signals were identified using adaptive minimum cross-entropy thresholding. The minimum cross-entropy method segments images into background and foreground by testing every possible threshold value and comparing the pixel intensity distributions on either side of the threshold. It selects the threshold where the intensity variation within the background and foreground is minimized, while the difference between the two groups is maximized, ensuring clear separation. Through adaptive thresholding, this method is applied to small subregions of the image, allowing for locally optimized threshold values in images with non-uniform intensity distributions. In situ signals were shrunk to a uniform diameter of 3 px to 5 px with local intensity maxima at the object center. For in situ signal decoding, the “RelateObjects” module was deployed for each utilized dual-color code to identify colocalized signals. This also identified and excluded false positive in situ signals that were only visible in one channel or did not correspond to any of the color codes used. In areas with high density of in situ signals, overlapping transcript-spots can lead to decoding problems and spurious calling of multiple transcripts. To minimize this effect, transcripts with expected high expression were used to mask potential false-positive transcripts in a multi-level process. VIM and KRT were used to mask AR-FL, i.e. AR-FL signals that overlapped with VIM and/or KRT were removed. VIM, KRT, AR-FL, and PSA were used to mask EPCAM, PSMA, AR-V7, pooled neuroendocrine markers NE, SLFN11, and DLL3. The “RelateObjects” module was also used to assign the decoded in situ signals to appointed cells. Three sources of false-positive in situ signals were observed. These sources were identified by additional CellProfiler modules and used to mask potentially false-positive in situ signals. First, objects with high autofluorescence in multiple channels were identified if detectable in both the Cy5 and TexasRed background scan. Second, colocalized in situ signals that were detectable in > 4 channels, were defined as unspecific signals. Third, empty image areas at the borders of the background scans resulting from image registration. For identification of nuclei, in situ signals, highly autofluorescent background objects, and, where applicable, propagated cell borders, we manually adjusted the threshold correction factor and lower bounds on threshold for each channel and sample, to fine-tune the calculated thresholds and to prevent extreme or invalid thresholds. The in situ signal count for each cell was exported to a csv spreadsheet and the results were visualized using the “OverlayOutlines” module. When analyzing patient samples, cells were classified directly in CellProfiler based on their in situ signal counts, using the “ClassifyObjects” module and a model trained on control samples in CellProfiler Analyst version 3 (Broad Institute of MIT and Harvard) [48].

#### Machine learning-based classification

To create a training and test dataset for the CellProfiler Analyst Classifier tool, 6 blood samples of healthy controls without (*n* = 3) or with (*n* = 3) spiked-in VCaP and PC-3 cells were enriched for CTCs and transcripts were visualized by in situ hybridization. After image analysis in CellProfiler, the full dataset was annotated by expert evaluation. Cells were categorized in 5 classes: CTCs (in situ signals detected for epithelial and/or prostate-specific markers), PBMCs (in situ signals detected for hematopoietic markers), artefacts (objects without nucleus, e.g. dust particles), in situ false-positive cells (in situ signals were detected by CellProfiler, but were rated as unspecific by the expert, e.g. due to high autofluorescence), and in situ negative cells (no in situ signals detectable by CellProfiler). The dataset was split into a training and test dataset. For the training dataset, 500 PBMCs were randomly chosen from the samples without spiked-in tumor cells. 200 CTCs were chosen randomly from the samples with spiked-in tumor cells. For the remaining classes, 100 cells each were randomly chosen from all samples. Cells with over- or undersegmentation of nuclei, and cells with both true-positive and false-positive in situ signals were not included in the training dataset. The training dataset was used in CellProfiler Analyst to train a random forest classifier, based on the decoded in situ signals per cell. The classifier was then evaluated in the test dataset. The classifier model was saved and integrated in the CellProfiler pipelines for image analysis and classification of liquid biopsy samples. These patient samples were subjected to expert revision to identify false-positive or false-negative CTCs and, where necessary, correct the RCP counts in CTCs.

#### Analysis of tissue sample

Original and background scan images were converted to TIF file format (LZW compression) in reduced resolution by 2 × 2 binning. Registration of original scan and background images was performed in a two-step process, similar to the CTC samples. Using pyStackReg, a pre-alignment of large image tiles (26,040 × 26,040 px) was followed by a second alignment of small image tiles (3000 × 3000 px), which were then used for CellProfiler analysis. In situ signals were segmented and decoded as described for CTC samples, with a few exceptions. The object size was adjusted to the reduced image resolution, no background subtraction was necessary, and highly autofluorescent background objects were identified based on the Atto425 and Atto488 background scans. Cell segmentation and assignment of decoded in situ signals, as well as the creation of a virtually stained H&E image, was done as described by Sallinger et al. [45]. Results were visualized using TissUUmaps (version 3.2.1.9) and the Points2Regions plugin (version 0.6.6) [49, 50]. To visualize clustering of coarse regions with Points2Regions, we set the pixel size and smoothing parameters to 20. At the single-cell level, we used hierarchical clustering via the Python library seaborn, on 10,000 cells that were randomly chosen from 10,000 × 10,000 px regions of non-neoplastic and neoplastic tissue, each [51].

### AdnaTest ProstateCancerSelect AR-V7

The AdnaTest ProstateCancerSelect AR-V7 (QIAGEN, Hilden, Germany) was used according to manufacturer’s guidelines as described previously [39]. In short, 5 ml of whole blood collected in PAXgene Blood ccfDNA Tubes (PreAnalytiX, Hombrechtikon, Switzerland) were used for immunomagnetic enrichment of CTCs. mRNA was isolated from the lysate of pre-enriched CTCs, and cDNA (20 µl) created by reverse transcription. The cDNA was then subjected to preamplification PCR in triplicates (6.25 µl cDNA per reaction). Finally, qRT-PCR was performed to detect CD45, GAPDH, PSA, PSMA, AR, and AR-V7. Patient samples were considered positive for the respective marker if it was detected in at least one of the cDNA triplicates.

### Data analysis and visualization

A number of Python libraries, including numpy and pandas, were used for data analysis and visualization [52–54]. Median and interquartile range (IQR) of CoDuCo in situ signals per cell (RCPs/cell) were calculated using the Python library pandas. Shapiro–Wilk test (scipy library) and Q-Q-plots (statsmodels library) were used to test data for normal distribution [55, 56]. As the data were not normally distributed, Kruskal–Wallis test (scipy) was performed to find significant differences, followed by pairwise comparisons using Dunn’s test (scikit_posthocs library), with p-value adjustment for multiple comparisons using the Benjamini and Hochberg method [57] and the resulting q-values were reported, with q-values < 0.05 considered statistically significant. To evaluate the performance of the random forest classifier for CTC detection, the sklearn library was used to calculate confusion matrix, multilabel confusion matrix, precision (positive predictive value), recall (sensitivity), f1-score, and support [58]. Furthermore, specificity and Matthews Correlation Coefficient were calculated [59, 60]. The Python libraries plotly, matplotlib, and seaborn were used to create plots and figures, which were finalized in Inkscape [51, 61]. PyCharm and Jupyter were used for Python projects and virtualenv and conda for managing virtual environments [62].

## Results

### Decoding of CoDuCo in situ signals

In situ PLP hybridization can be used to visualize transcripts in CTCs. Aiming to increase the number of detectable transcripts, we developed a novel staining approach. With a conventional approach, a seven-channel fluorescence microscope can detect a maximum of six mRNA markers along with DAPI-stained nuclei. To address this limitation, we introduced the CoDuCo approach, which employs a two-color code for in situ signal detection. Given six fluorescence channels for in situ signals (*n* = 6) and a two-color code (k = 2), the total number of distinct combinations is $\left(\genfrac{}{}{0pt}{}{\text{n}}{{\text{k}}}\right)= \text{15}$.

**Fig. 1:**
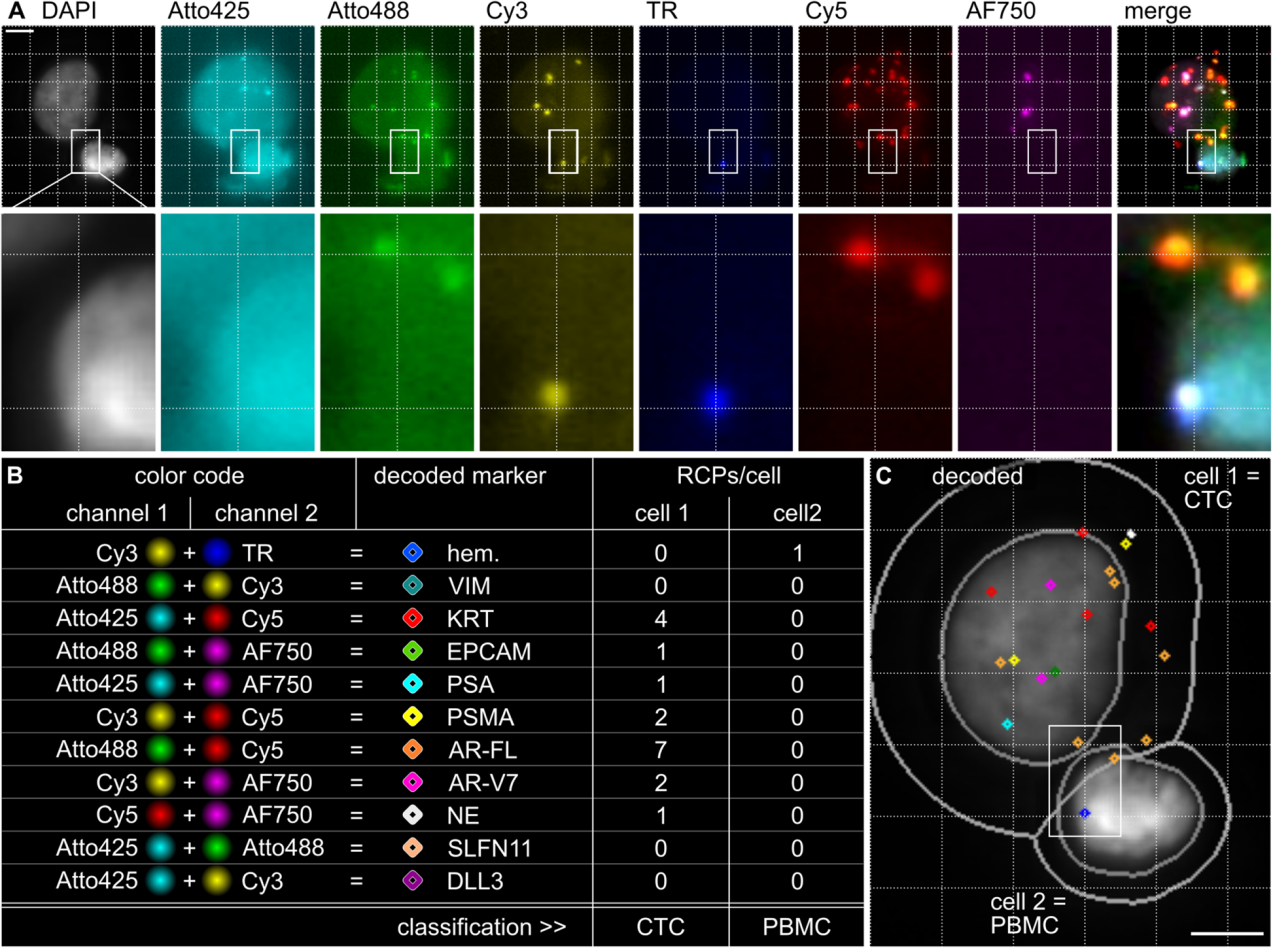
Decoding CoDuCo *in situ* signals to identify CTCs and PBMCs. **A** Pseudocolored images of two cells with DAPI-stained nuclei and CoDuCo *in situ* signals in 6 channels. The dotted 5 μm grid is a visual aid, guiding the eye to recognize colocalized *in situ* signals. One region is highlighted and shown in higher magnification. **B** Decoding scheme for all markers and their expression levels [RCPs/cell] in the two cells. E.g. *in situ* signals that are visible at the exact same x and y coordinates in the Cy3 and TR (TexasRed) channel, can be decoded as hematopoietic (hem.) markers. The respective colored diamond-shapes are used in **C** to visualize decoded *in situ* signals, together with gray outlines of nuclei and cell borders on the DAPI image. PBMCs can be identified based on their expression of hematopoietic markers, and CTCs can be identified based on the expression of epithelial (KRT and EPCAM) and/or prostate-specific markers (PSA, PSMA, AR-FL, AR-V7). Scale bar 5 μm

We had noticed bleed-through of Cy5 into the TexasRed channel. To minimize the risk for decoding errors, we made a strategic choice and opted to use TexasRed exclusively for detecting hematopoietic markers in PBMCs, in combination with Cy3. This decision resulted in a total of 11 unique color combinations. Figure 1 provides a summary of all markers and their corresponding color codes. Additionally, it illustrates how a tumor cell and a PBMC were identified based on the decoded in situ signals. To cover a broad range of PBMCs, we used a panel of five hematopoietic markers, that were all detected by the TexasRed + Cy3 combination, namely, PTPRC (CD45), ITGAM (CD11B), FCGR3A and FCGR3B (CD16), CD4, and ITGB2 (CD18). Similar to the pooled hematopoietic markers, we detected pooled KRT markers (KRT8, KRT18, and KRT19) using the Atto425 + Cy5 code, and pooled neuroendocrine markers NE (SYP, CHGA, and NCAM1) using Cy5 + AF750. The remaining color codes were used to detect VIM (Atto488 + Cy3), EPCAM (Atto488 + AF750), PSA (Atto425 + AF750), PSMA (Cy3 + Cy5), AR-FL (Atto488 + Cy5), AR-V7 (Cy3 + AF750), SLFN11 (Atto425 + Atto488), and DLL3 (Atto425 + Cy3).

### Validation of CoDuCo for CTC characterization

**Fig. 2:**
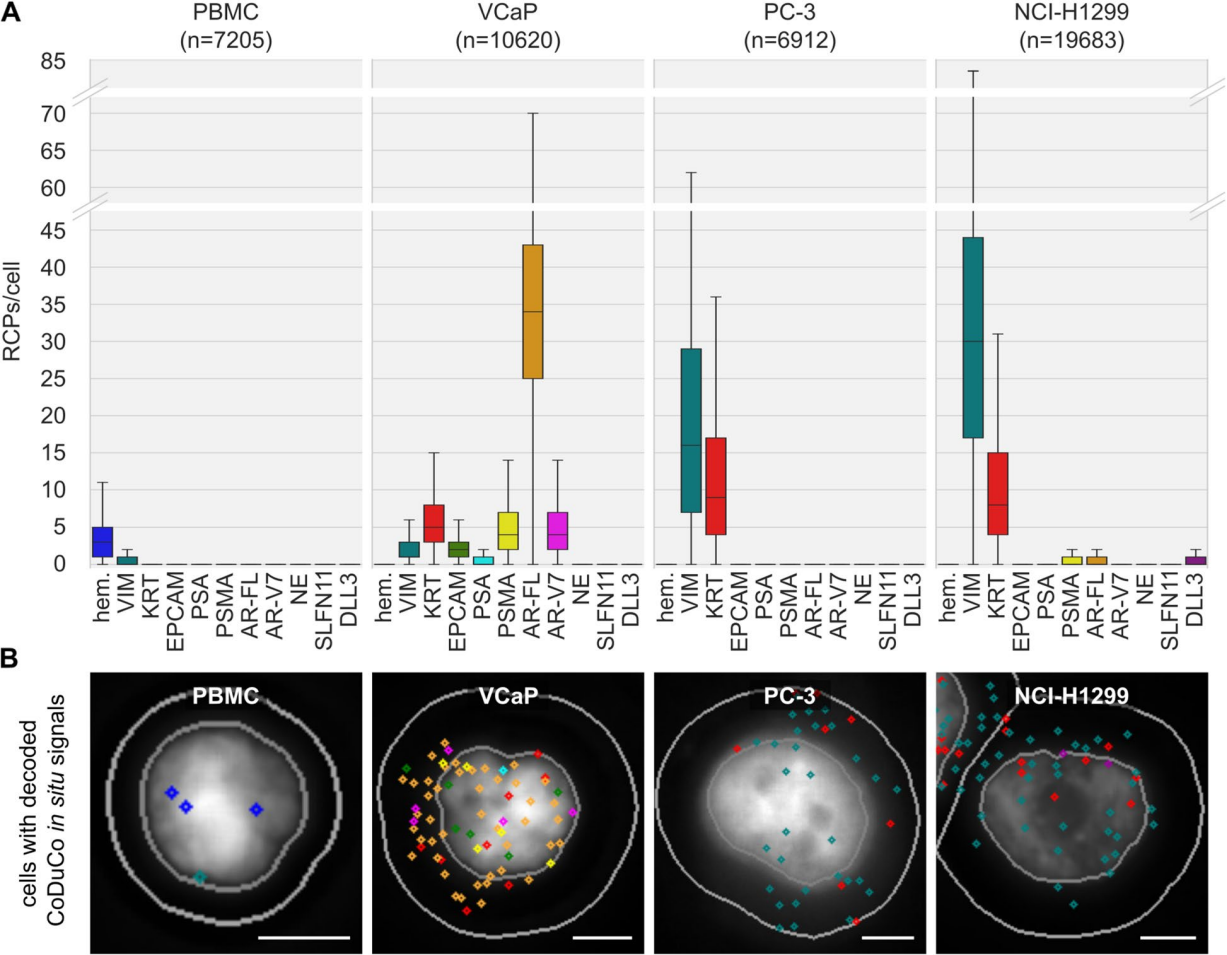
CoDuCo *in situ* assay visualizes distinctive expression profiles in PBMCs, VCaP, PC-3, and NCI-H1299 cells. **A** The number of *in situ* signals per cell (RCPs/cell) is visualized for each marker and cell type/cell line. Outliers are not shown in the boxplots. **B** Exemplary images for each cell type, showing DAPI staining, nucleus and cell border outlines (gray), and decoded *in situ* signals as diamond shapes with the same color scheme as for the boxplots (e.g. blue diamond shapes represent hematopoietic markers). Scale bar 5 μm

To validate the novel CoDuCo in situ assay, we applied it to healthy control PBMCs, PC cell lines VCaP and PC-3, and non-small cell lung cancer cell line NCI-H1299. We detected CoDuCo in situ signals in 89% of PBMCs (*n* = 7205 cells), 99% of VCaP cells (*n* = 10,620 cells), 93% of PC-3 cells (*n* = 6912 cells), and 100% of NCI-H1299 cells (*n* = 19,683). The assay revealed distinctive gene expression profiles for PBMCs, VCaP cells, PC-3 cells, and NCI-H1299 cells, as summarized in Fig. 2 and Table 1. Pairwise comparison revealed significant differences (q ≤ 0.05) in the expression of all markers between PBMCs and tumor cells, with the exception of SLFN11 and AR-V7 between PBMCs and PC-3 cells, and EPCAM, PSA, and PSMA between PBMCs and NCI-H1299 cells. Only for a small percentage it was not possible to differentiate between PBMCs and tumor cells using specific thresholds, since 1–8% of tumor cells expressed hematopoietic markers and 7% of PBMCs were positive for at least one epithelial or prostate-specific marker.

**Table 1:** CoDuCo in situ expression patterns in PBMCs and tumor cell lines VCaP, PC-3, and NCl-H1299

	**PBMC** (*n* = 7205)		**VCaP** (*n* = 10,620)		**PC-3** (*n* = 6912)		**NCI-H1299** (*n* = 19,683)		**q-value** PBMC versus		
Marker	Positivity [%]	Median (Q1, Q3) [RCPs/cell]	Positivity [%]	Median (Q1, Q3) [RCPs/cell]	Positivity [%]	Median (Q1, Q3) [RCPs/cell]	Positivity [%]	Median (Q1, Q3) [RCPs/cell]	VCaP	PC-3	NCI-H1299
hem	86	3 (1–5)	2	0 (0–0)	8	0 (0–0)	1	0 (0–0)	**0.000**	**0.000**	**0.000**
VIM	32	0 (0–1)	75	1 (1–3)	92	16 (7–29)	99	30 (17–44)	**0.000**	**0.000**	**0.000**
KRT	4	0 (0–0)	94	5 (3–8)	90	9 (4–17)	97	8 (4–15)	**0.000**	**0.000**	**0.000**
EPCAM	0	0 (0–0)	79	2 (1–3)	21	0 (0–0)	0	0 (0–0)	**0.000**	**0.000**	0.848
PSA	0	0 (0–0)	44	0 (0–1)	3	0 (0–0)	1	0 (0–0)	**0.000**	**0.000**	0.501
PSMA	1	0 (0–0)	93	4 (2–7)	15	0 (0–0)	28	0 (0–1)	**0.000**	**0.000**	**0.000**
AR-FL	1	0 (0–0)	99	34 (25–43)	20	0 (0–0)	32	0 (0–1)	**0.000**	**0.000**	**0.000**
AR-V7	1	0 (0–0)	90	4 (2–7)	1	0 (0–0)	1	0 (0–0)	**0.000**	0.906	0.673
NE	4	0 (0–0)	12	0 (0–0)	3	0 (0–0)	5	0 (0–0)	**0.000**	**0.003**	**0.014**
SLFN11	7	0 (0–0)	16	0 (0–0)	6	0 (0–0)	14	0 (0–0)	**0.000**	0.415	**0.000**
DLL3	1	0 (0–0)	14	0 (0–0)	6	0 (0–0)	26	0 (0–1)	**0.000**	**0.000**	**0.000**
total	89	3 (2–6)	99	55 (41–69)	93	28 (15–48)	100	41 (24–61)	**0.000**	**0.000**	**0.000**

Positivity = percentage of cells with ≥ 1 RCP/cell for the respective marker

q-value from pairwise comparison between PBMCs and respective cancer cell lines using Dunn’s Test with Benjamini/Hochberg correction. q-values < 0.05 are highlighted in bold font

hem.: pooled hematopoietic markers (PTPRC, ITGAM, FCGR3, CD4, ITGB2); KRT: pooled KRT markers (KRT8, KRT18, KRT19); NE: pooled neuroendocrine markers (SYP, CHGA, NCAM1)

In detail, pooled hematopoietic markers were expressed with an overall median of 3 RCPs/cell (IQR 1–5) in PBMCs. These markers were detected in 86% of PBMCs. 2% of VCaP, 8% of PC-3, and 1% of NCI-H1299 cells were positive for pooled hematopoietic markers, but with very low overall expression levels (median 0 RCPs/cell, IQR 0–0). The assay detected low expression of epithelial and prostate-specific transcripts (KRT, EPCAM, PSA, PSMA, AR-FL, and AR-V7) in up to 4% of PBMCs, each (overall median 0 RCPs/cell, IQR 0–0). All epithelial and prostate-specific markers were expressed in VCaP cells. PSA had the lowest expression with a median of 0 RCPs/cell (IQR 0–1) and positivity in 44% of cells. AR-FL had the highest expression with a median of 34 RCPs/cell (IQR 25–43) and positivity in 99% of cells. 90% of PC-3 cells expressed KRT, with a median overall expression of 9 RCPs/cell (IQR 4–17). Low expression of EPCAM and prostate-specific markers was detected in up to 21% of PC-3 cells, each (overall median 0 RCPs/cell, IQR 0–0). 97% of NCI-H1299 cells expressed KRT, with a median overall expression of 8 RCPs/cell (IQR 4–15). Low expression of PSA and AR-V7 was detected in 1% of NCI-H1299 cells, each (overall median 0 RCPs/cell, IQR 0–0). PSMA and AR-FL were detected in 28% and 32% of NCI-H1299 cells, respectively (overall median 0 RCPs/cell, IQR 0–1). VIM was detected in 32% of PBMCs, with a median overall expression of 0 RCPs/cell (IQR 0–1), in 75% of VCaP cells, with a median of 1 RCPs/cell (IQR 1–3), in 92% of PC-3 cells, with a median of 16 RCPs/cell (IQR 7–29), and in 99% of NCI-H1299 cells, with a median of 30 RCPs/cell (IQR 17–44). Low expression of pooled neuroendocrine markers, SLFN11, and DLL3, was detected in 4%, 7%, and 1% of PBMCs, 12%, 16%, and 14% of VCaP cells, and 3%, 6%, and 6% of PC-3 cells, respectively, with median overall expression of 0 RCPs/cell (IQR 0–0). Neuroendocrine markers and SLFN11 were detected in 5% and 14% of NCI-H1299 cells, respectively (overall median 0 RCPs/cell, IQR 0–0), and DLL3 in 26% of NCI-H1299 cells (overall median 0 RCPs/cell, IQR 0–1).

### Classifier training and evaluation

**Fig. 3:**
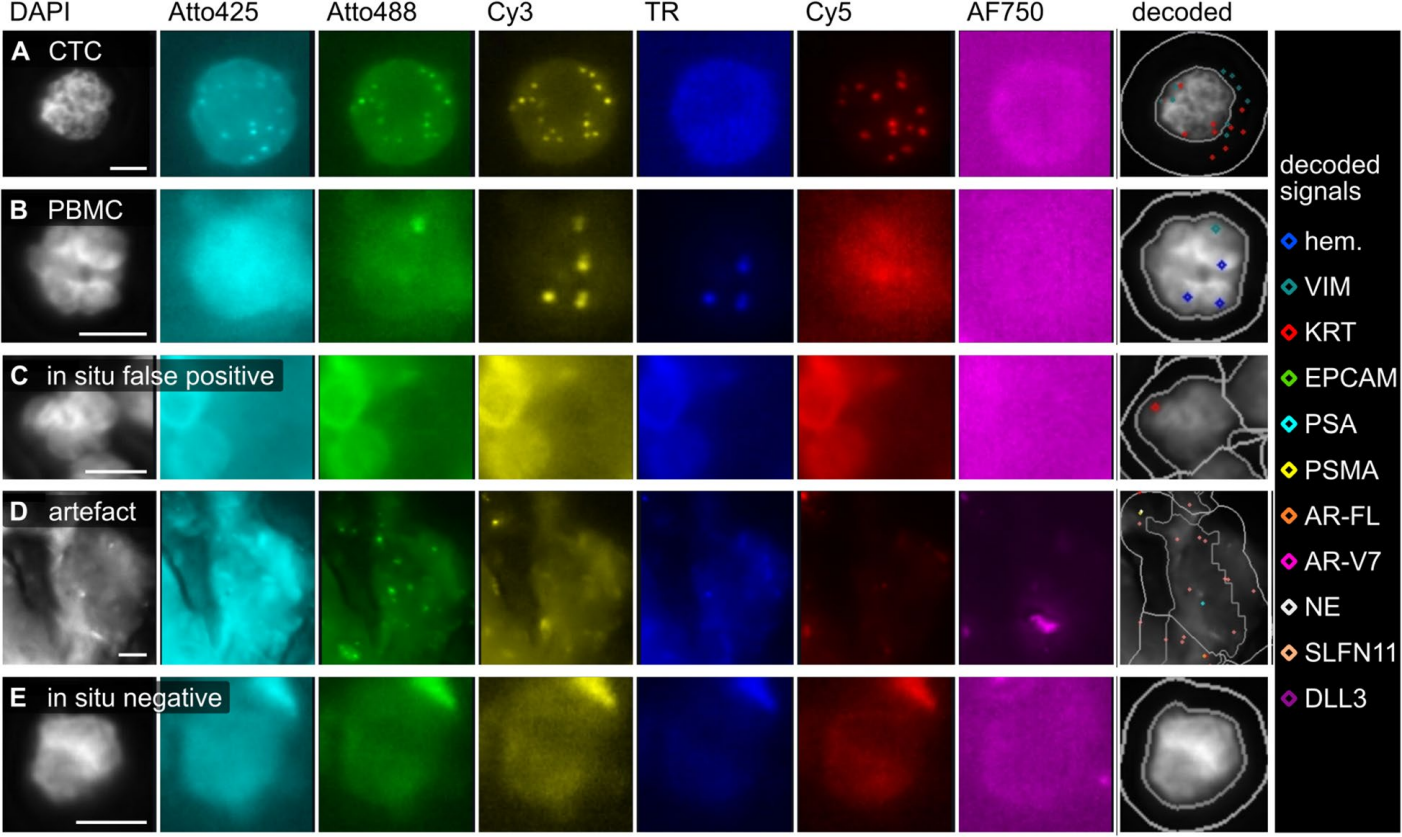
Annotation of the ground truth dataset based on the decoded *in situ* signals. **A**-**E** For each of the 5 classes, one example is shown. **C** Objects with false-positive in situ signals (e.g. due to high autofluorescence) were classified as *in situ* false-positive (intact nuclear morphology) or **D** artefact (e.g. dust particles). **E** Objects without decoded *in situ* signals were classified as in situ negative. Scale bar 5 μm

To create a ground truth for the classifier, a dataset of 137,871 cells was used, derived from 6 blood samples of healthy controls without (*n* = 3) or with (*n* = 3) spiked-in VCaP and PC-3 cells. Spiked blood samples were processed using the CytoGen Smart Biopsy Cell Isolator, transcripts were visualized and counted by CoDuCo in situ hybridization and CellProfiler image analysis, and all detected cells in the dataset were manually classified into the following 5 classes through expert evaluations: CTCs, PBMCs, artefacts, in situ false-positive cells, and in situ negative cells (Fig. 3).

**Fig. 4:**
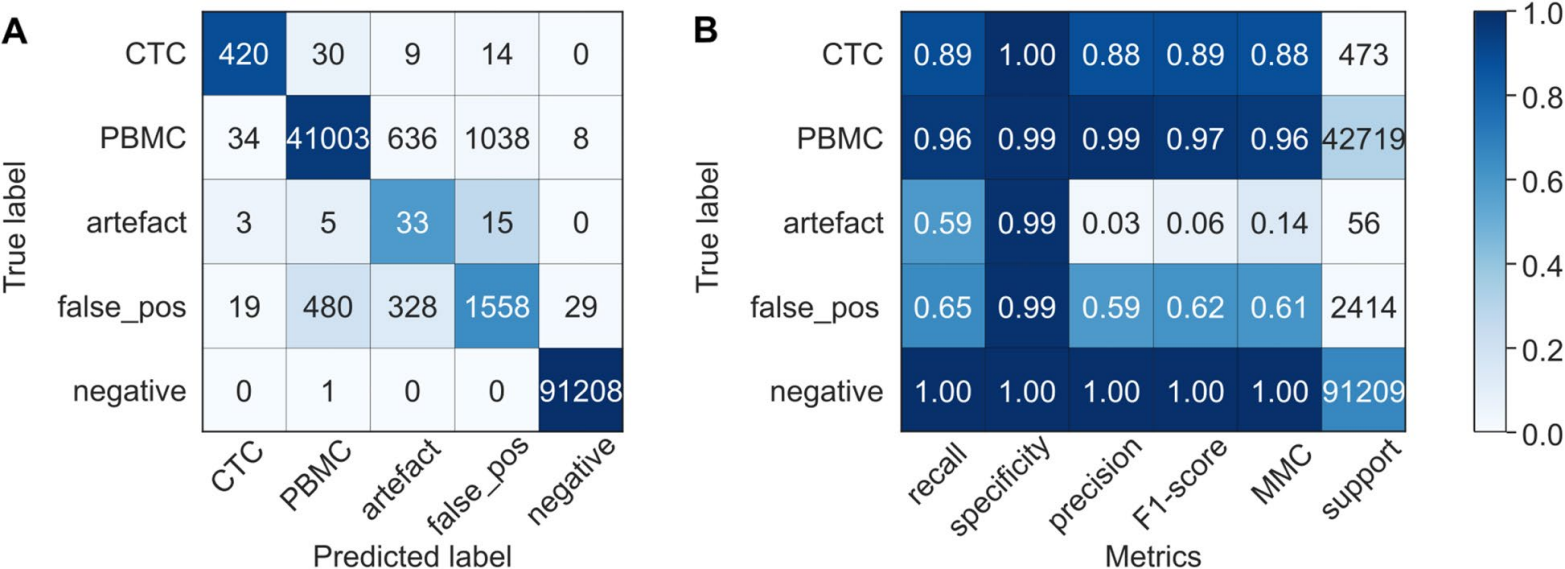
Classifier evaluation (test dataset). A random forest classifier was trained and tested on blood samples of healthy controls with and without spiked-in tumor cells. **A** Results of the classifier evaluation in the test dataset are summarized in a confusion matrix and **B** a classification report. In the confusion matrix, the colors indicate values normalized to the class support size (i.e. the number of actual occurrences in each group, as indicated in panel **B**), while the annotation shows non-normalized values. false_pos = cells without true-positive *in situ* signals; negative = cells with no *in situ* signals detected by CellProfiler analysis; recall = fraction of correctly identified positives; specificity = fraction of correctly identified negatives; precision = accuracy of positive predictions; F1-score = harmonic mean of precision and recall; MCC = Matthews Correlation Coefficient

The ground truth dataset of 137,871 cells was classified into the following classes: 673 CTCs (0.49%), 43,219 PBMCs (31.35%), 156 artefacts (0.11%), 2514 in situ false-positive cells (1.82%), and 91,309 in situ negative cells (66.23%). For sufficient representation of all classes in the training dataset, we randomly selected 1000 cells at a ratio of 2/5/1/1/1 (200 CTCs, 500 PBMCs, 100 of each of the remaining classes). After training a random forest classifier, we evaluated it in the remaining test dataset of 136,871 cells. Confusion matrix and evaluation metrics are visualized in Fig. 4. In the control samples, the classifier reached a high recall (0.89), precision (0.88), F1-score (0.89) and specificity (1.00) for CTCs.

### The classifier identified patient CTCs with a recall of 0.76 and specificity of 0.99

**Fig. 5:**
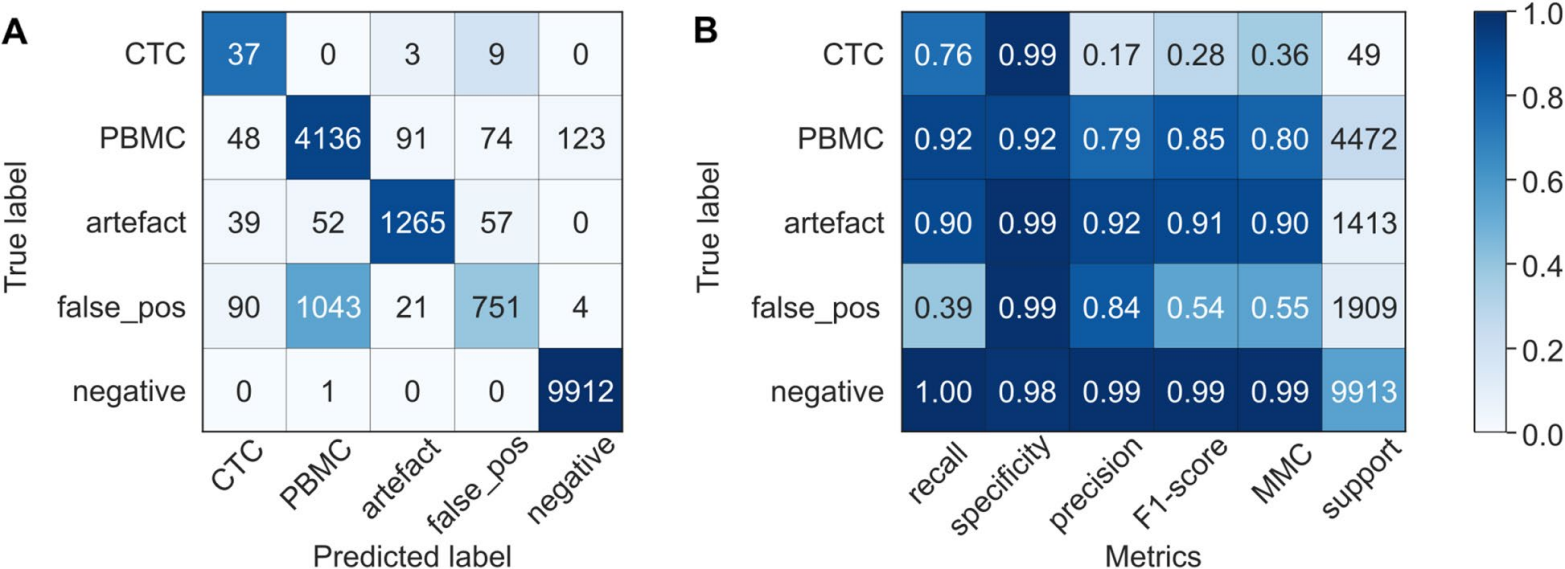
Classifier evaluation on three patient samples. A random forest classifier was trained on blood samples of healthy controls with and without spiked-in tumor cells. **A** Results of the classifier evaluation in the test dataset are summarized in a confusion matrix and **B** a classification report. In the confusion matrix, the colors indicate values normalized to the class support size (i.e. the number of actual occurrences in each group, as indicated in panel **B**), while the annotation shows non-normalized values. false_pos = cells without true-positive *in situ* signals; negative = cells with no *in situ* signals detected by CellProfiler analysis; recall = fraction of correctly identified positives; specificity = fraction of correctly identified negatives; precision = accuracy of positive predictions; F1-score = harmonic mean of precision and recall; MCC = Matthews Correlation Coefficient

Eventually, we tested the complete workflow, including blood collection, CTC enrichment, in situ hybridization, image analysis, cell classification, and expert revision, on three patient samples (PC-13, PC-15, PC-16). In total, 17,756 cells were detected by automated image analysis. During expert revision, 49 of them were identified as CTCs, of which 37 (76%) were also recognized by the classifier, resulting in a recall of 0.76 as visualized in Fig. 5. Among 17,707 non-CTCs, 177 false-positive CTCs were reported by the classifier, corresponding to a specificity of 0.99. With 37 true-positive and 177 false-positive CTCs, the classifier reached a precision of 0.17.

Comparative analysis of expression patterns between false-negative (24%) and true-positive (76%) CTCs revealed major differences (Table 2). In CTCs that were missed by the classifier, the total number of automatically detected in situ signals per cell was significantly decreased, with a median of 4 RCPs/cell (IQR 3–7) compared to a median of 35 RCPs/cell (IQR 18–67) in true-positive CTCs (q ≤ 0.0001). Furthermore, there was a significant decrease in expression levels of KRT (q ≤ 0.0001), PSA (q ≤ 0.001), AR-FL (q ≤ 0.001), and AR-V7 (q ≤ 0.05) in false-negative CTCs. 75% of false-negative CTCs showed no KRT expression, while KRT was expressed in 97% of true-positive CTCs. In KRT-negative CTCs, the classifier performance was significantly decreased, with a recall of 0.10 compared to 0.92 for KRT-positive CTCs.

**Table 2:** Comparison of expression patterns in patient CTCs that were missed or recognized by the classifier

Marker	False-negative CTCs (*n* = 12)		True-positive CTCs (*n* = 37)		q-value
	Positivity [%]	Median (Q1, Q3) [RCPs/cell]	Positivity [%]	Median (Q1, Q3) [RCPs/cell]	
hem	0	0 (0, 0)	11	0 (0, 0)	0.240
VIM	25	0 (0, 0)	32	0 (0, 1)	0.392
KRT	25	0 (0, 0)	97	5 (2, 10)	**0.000**
EPCAM	8	0 (0, 0)	22	0 (0, 0)	0.292
PSA	92	2 (1, 2)	97	10 (5, 39)	**0.000**
PSMA	8	0 (0, 0)	22	0 (0, 0)	0.263
AR-FL	58	1 (0, 2)	92	4 (2, 11)	**0.000**
AR-V7	8	0 (0, 0)	46	0 (0, 2)	**0.024**
NE	8	0 (0, 0)	11	0 (0, 0)	0.808
SLFN11	25	0 (0, 0)	30	0 (0, 1)	0.659
DLL3	33	0 (0, 1)	14	0 (0, 0)	0.167
total	100	4 (3, 7)	100	35 (18, 67)	**0.000**

Positivity = percentage of cells with ≥ 1 RCP/cell for the respective marker

q-value from pairwise comparison between false-negative and true-positive CTCs using Dunn’s Test with Benjamini/Hochberg correction. q-values < 0.05 are highlighted in bold font

hem.: pooled hematopoietic markers (PTPRC, ITGAM, FCGR3, CD4, ITGB2); KRT: pooled KRT markers (KRT8, KRT18, KRT19); NE: pooled neuroendocrine markers (SYP, CHGA, NCAM1)

### CoDuCo revealed interpatient CTC heterogeneity and captured neuroendocrine CTCs and CTC-clusters

**Fig. 6:**
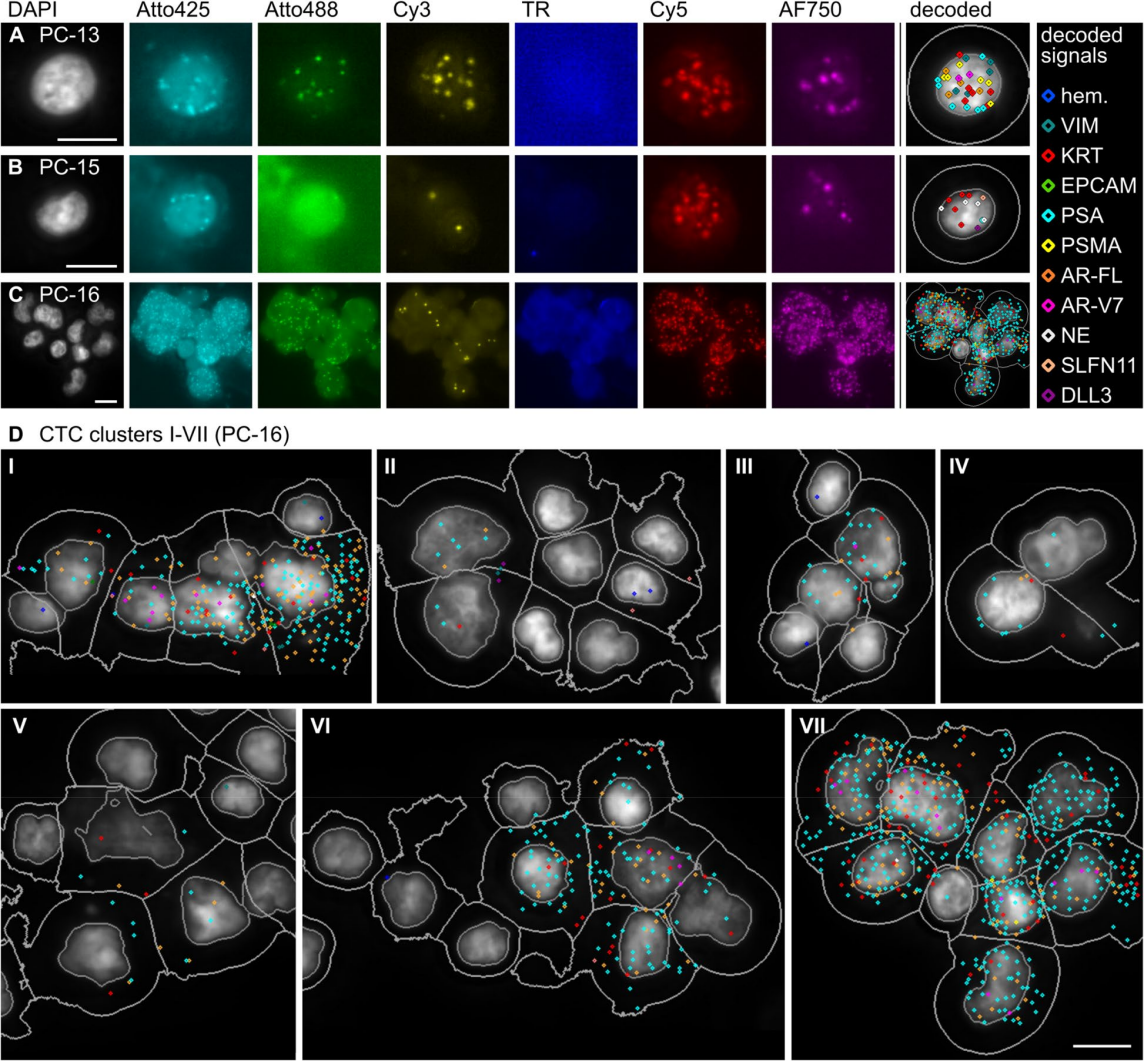
Interpatient heterogeneity highlighted by exemplary images of patient CTCs. **A**-**C** Normalized pseudocolored fluorescence images and overlay images showing the DAPI channel with outlines of nuclei, cell borders, and decoded *in situ* signals as detected by CellProfiler analysis. **D** Overlay images with decoded *in situ* signals in seven CTC-clusters that were found in PC-16. Only cluster IV and VII consisted exclusively of CTCs. The other clusters were mixed and contained PBMCs and/or in situ negative cells as well as CTCs. Scale bar 10 μm

Interpatient heterogeneity was observed regarding CTC count, presence of CTC clusters, as well as expression patterns. Exemplary images of patient CTCs are depicted in Fig. 6. We found 8 CTCs in sample PC-13, 3 CTCs in PC-15, and 38 CTCs in PC-16. In PC-16, most CTCs (26 of 38) were found in clusters of up to 9 CTCs (Fig. 6D), while no CTC-clusters were found in the other samples. The interpatient differences in CTC numbers and their expression level are summarized in Table 3. PC-13 CTCs were characterized by high VIM and KRT expression and medium to low expression of prostate-specific markers, such as PSMA and AR-V7. PC-16 CTCs showed high expression of PSA, medium expression of AR-FL, low expression of KRT, and very low overall expression of VIM, PSMA, and AR-V7. In contrast, CTCs in PC-15 expressed no prostate-specific markers but expressed KRT and neuroendocrine markers at a high level. Pairwise comparison between patient samples revealed that VIM and PSMA expression was significantly increased in PC-13 (q ≤ 0.05), PSA expression was significantly increased in PC-16, and the expression of pooled neuroendocrine markers, SLFN11, and DLL3 was significantly increased in PC-15. No significant difference in KRT expression was detected. In detail, the median number of RCPs/cell in CTCs of PC-13 was 8 for VIM (IQR 4–15), 8 for KRT (IQR 2–14), 2 for PSA (IQR 0–8), 4 for PSMA (IQR 2–5), 2 for AR-FL (IQR 1–3), and 0 for AR-V7 (IQR 0–1). In CTCs of PC-15, the median number of RCPs/cell was 7 for KRT (IQR 6–8), 0 for EPCAM (IQR 0–1), 7 for pooled neuroendocrine markers (IQR 6–10), 2 for SLFN11 (IQR 1–3), and 1 for DLL3 (IQR 0–2). In CTCs of PC-16, the median number of RCPs/cell was 2 for KRT (IQR 1–5), 10 for PSA (IQR 4–38), 4 for AR-FL (IQR 1–10), and 0 for AR-V7 (IQR 0–1).

**Table 3:** Interpatient comparison of CTC expression patterns

Marker	PC-13 CTCs (*n* = 8)		PC-15 CTCs (*n* = 3)		PC-16 CTCs (*n* = 38)		q-values		
	Positivity [%]	Median (Q1, Q3) [RCPs/cell]	Positivity [%]	Median (Q1, Q3) [RCPs/cell]	Positivity [%]	Median (Q1, Q3) [RCPs/cell]	PC-13 & PC-15	PC-13 & PC-16	PC-15 & PC-16
hem	0	0 (0–0)	0	0 (0–0)	3	0 (0–0)	1.000	1.000	1.000
VIM	88	8 (4–15)	33	0 (0–0)	3	0 (0–0)	**0.031**	**0.000**	0.268
KRT	75	8 (2–14)	100	7 (6–8)	76	2 (1–5)	0.628	0.195	0.195
EPCAM	25	0 (0–0)	33	0 (0–1)	8	0 (0–0)	0.591	0.319	0.319
PSA	62	2 (0–8)	0	0 (0–0)	100	10 (4–38)	0.214	**0.026**	**0.010**
PSMA	88	4 (2–5)	0	0 (0–0)	5	0 (0–0)	**0.001**	**0.000**	0.845
AR-FL	75	2 (1–3)	0	0 (0–0)	89	4 (1–10)	0.158	0.158	**0.023**
AR-V7	50	0 (0–1)	0	0 (0–0)	32	0 (0–1)	0.394	0.506	0.394
NE	0	0 (0–0)	100	7 (6–10)	0	0 (0–0)	**0.000**	1.000	**0.000**
SLFN11	25	0 (0–0)	67	2 (1–3)	3	0 (0–0)	**0.036**	0.072	**0.001**
DLL3	12	0 (0–0)	67	1 (0–2)	0	0 (0–0)	**0.001**	0.194	**0.000**
**total**	100	39 (21–50)	100	20 (16–22)	100	16 (7–54)	0.955	0.955	0.971

Positivity = percentage of cells with ≥ 1 RCP/cell for the respective marker

q-value from pairwise interpatient comparison using Dunn’s Test with Benjamini/Hochberg correction. q-values < 0.05 are highlighted in bold font

hem.: pooled hematopoietic markers (PTPRC, ITGAM, FCGR3, CD4, ITGB2); KRT: pooled KRT markers (KRT8, KRT18, KRT19); NE: pooled neuroendocrine markers (SYP, CHGA, NCAM1)

### CoDuCo in situ revealed intrapatient CTC heterogeneity

**Fig. 7:**
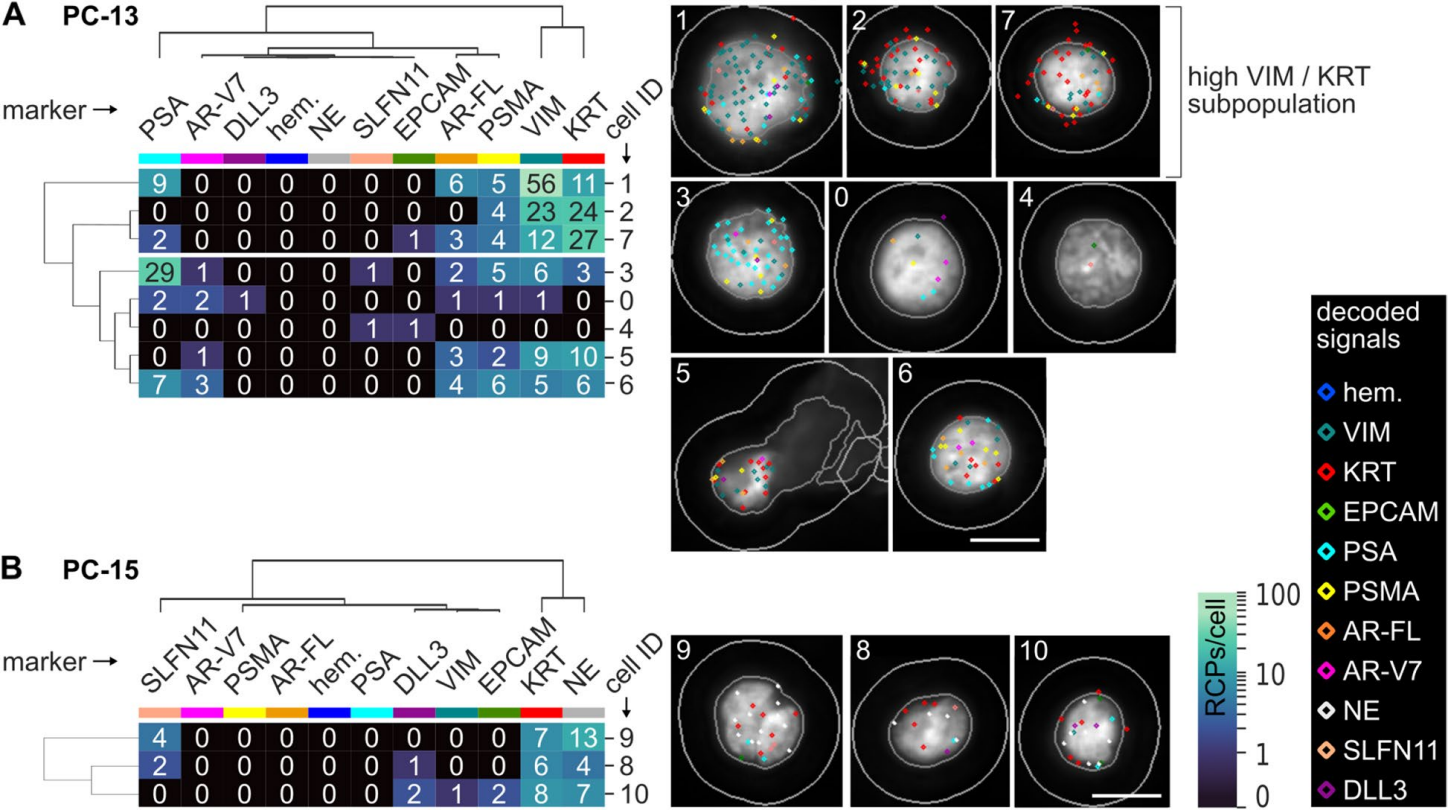
CTCs of patients **A** PC-13 and **B** PC-15. Dendrograms as well as the order of cells (rows) and markers (columns) are based on hierarchical clustering of CTCs. The number of in situ signals/cell is visualized in the heatmaps and corresponding CTC thumbnails. The thumbnails show the DAPI image with cell ID number, outlines of nuclei, cell borders, and decoded in situ signals as detected by CellProfiler analysis. The colors of decoded in situ signals is indicated for each marker on top of the clustermap. For instance, red dots are KRT transcripts. We found heterogeneous subpopulations of CTCs (high VIM/KRT and PSA/AR-V7, respectively) in patient PC-13 (**A**) and neuroendocrine CTCs in PC-15 (**B**). Pools of 3-5 genes were used for hematopoietic hem. (PTPRC, ITGAM, FCGR3A&B, CD4, ITGB2), KRT (KRT8, KRT18, KRT19), and neuroendocrine NE markers (SYP, CHGA, NCAM1). Scale bar 10 μm

**Fig. 8:**
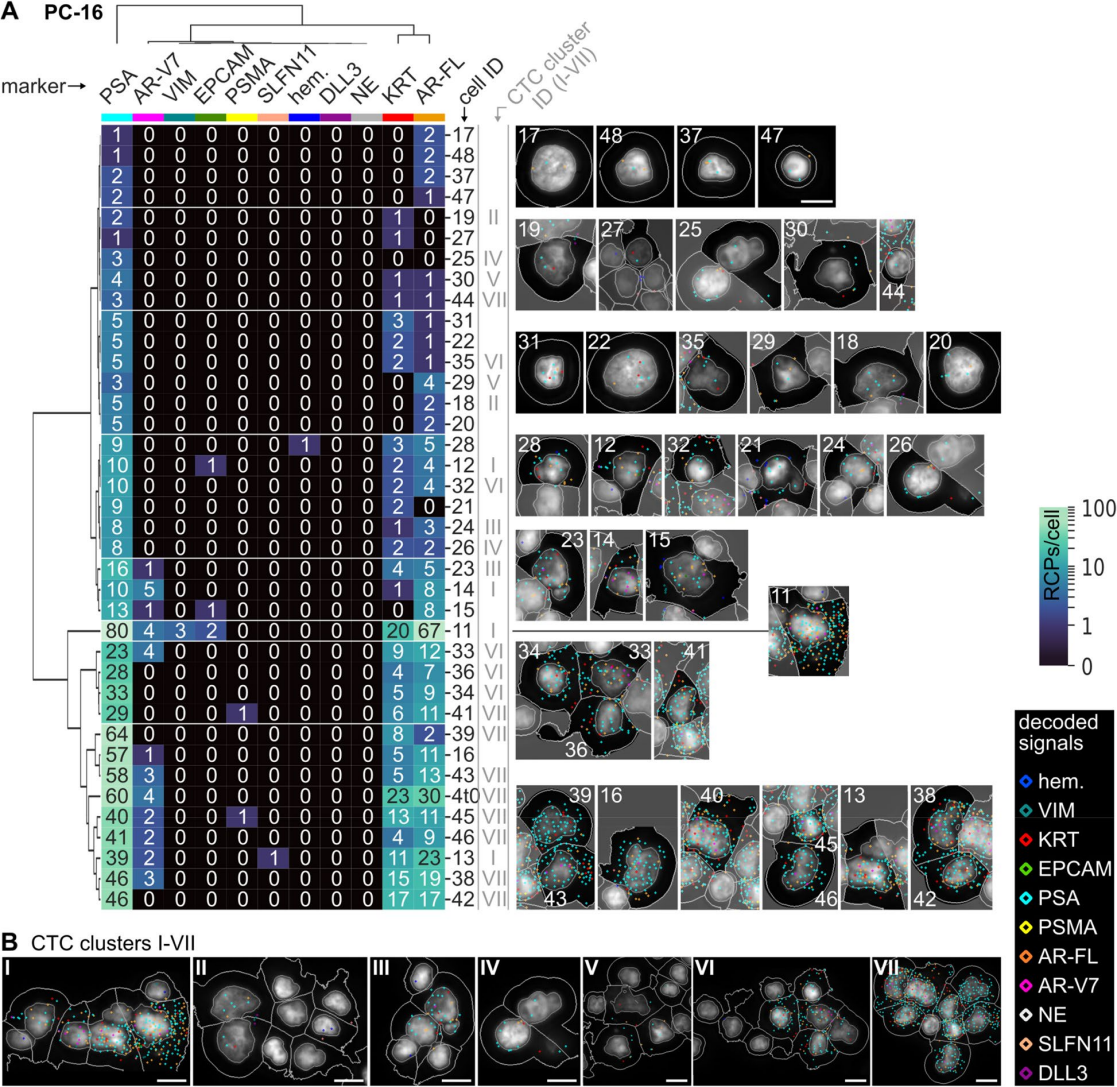
CTCs of patient PC-16. **A** Dendrograms as well as the order of cells (rows) and markers (columns) are based on hierarchical clustering of CTCs. The number of in situ signals/cell is visualized in the heatmaps and corresponding CTC thumbnails. The thumbnails show the DAPI image with cell ID number, outlines of nuclei, cell borders, and decoded in situ signals as detected by CellProfiler analysis. The colors of decoded in situ signals is indicated for each marker on top of the clustermap. **B** Overview of 7 CTC-clusters (IDs I-VII) detected in PC-16. Pools of 3-5 genes were used for hematopoietic hem. (PTPRC, ITGAM, FCGR3A&B, CD4, ITGB2), KRT (KRT8, KRT18, KRT19), and neuroendocrine NE markers (SYP, CHGA, NCAM1). Scale bar 5 μm

On a single-cell level, individual CTCs showed very heterogeneous expression patterns as visualized in the clustermaps and CTC images in Fig. 7 and Fig. 8. In PC-13, multiple CTCs had remarkably high coexpression of VIM and KRT in the absence of AR-V7, while others had more prominent expression of prostate-specific markers, especially PSA and AR-V7 (Fig. 7A). The three CTCs that were detected in patient sample PC-15 all showed coexpression of KRT and neuroendocrine markers (Fig. 7B). In PC-16, all CTCs expressed PSA, but the expression level ranged from 1 RCP/cell to 80 RCPs/cell. Similarly, most CTCs were positive for KRT and AR-FL, with expression levels ranging from 0–20 RCPs/cell and 0–67 RCPs/cell, respectively. Furthermore, 12/38 CTCs expressed AR-V7. While the median overall expression of AR-V7 was 0 RCPs/cell (IQR 0–1), in the subset of AR-V7-positive CTCs, the median expression was 3 RCPs/cell (IQR 2–4) (Fig. 8).

### CoDuCo in situ showed high concordance with clinical parameters and AdnaTest

**Fig. 9:**
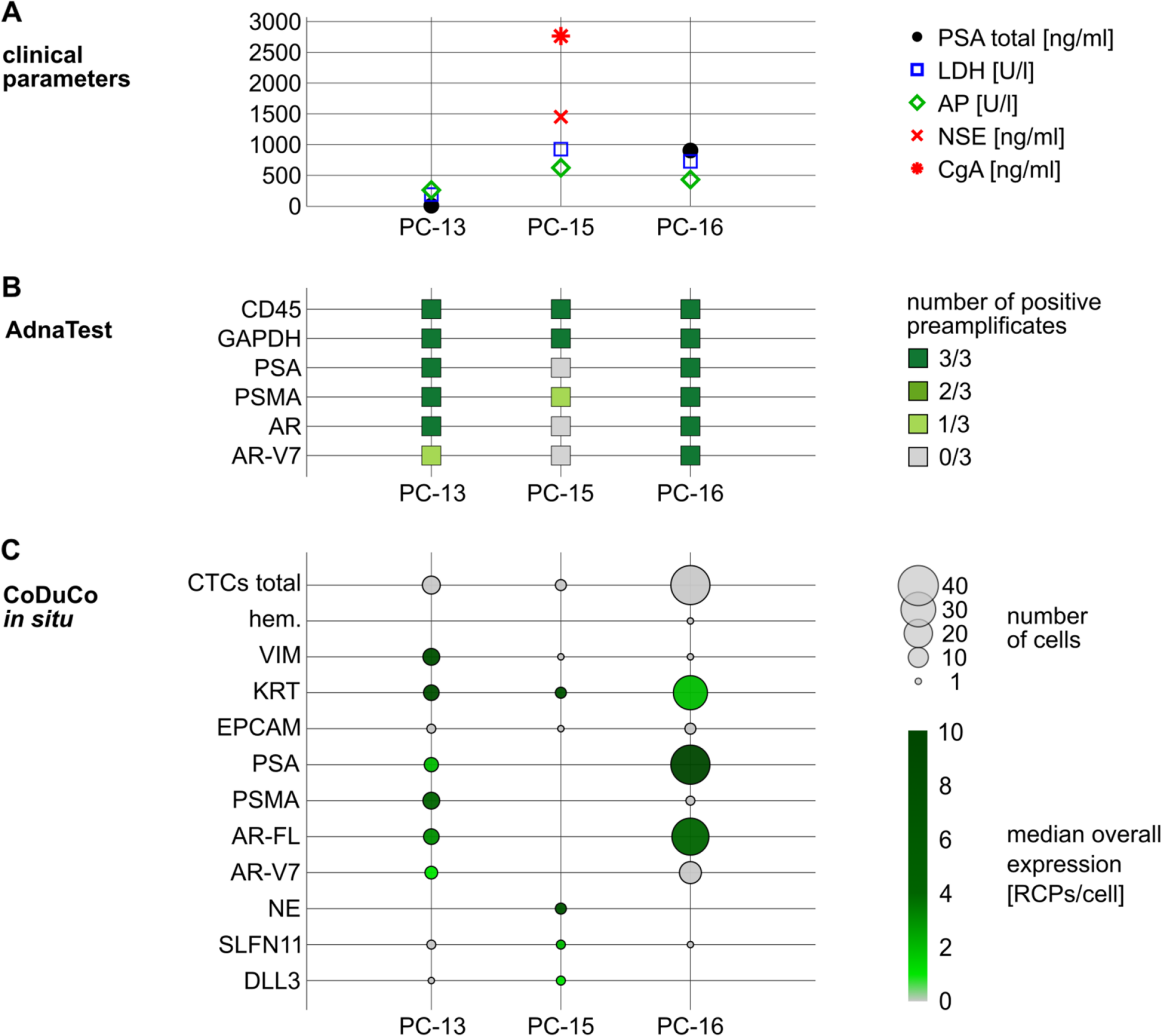
Clinical parameters and results of CTC-analyses by AdnaTest and CoDuCo *in situ* assay. Data is shown for patients PC-13, PC-15, and PC-16. **A** Blood levels of total PSA [ng/ml], lactate dehydrogenase (LDH) [U/l], alkaline phosphatase (AP) [U/l], neuron-specific enolase (NSE) [ng/ml], and chromogranin A (CgA) [ng/ml], where available. **B** AdnaTest results. For each sample, 3 preamplifications of isolated and reverse-transcribed RNA were performed for full coverage. The absence or presence of PSA, PSMA, AR (=AR-FL), and AR-V7 transcripts is visualized by colored squares (dark green = detected in all preamplificates; gray = not detected). **C** Bubble plot visualizing the number (bubble size) and median overall expression levels (color scale) of *in situ* analyzed CTCs (dark green = high expression; gray = low expression). hem.: pooled hematopoietic markers (PTPRC, ITGAM, FCGR3, CD4, ITGB2); KRT: pooled KRT markers (KRT8, KRT18, KRT19); NE: pooled neuroendocrine markers (SYP, CHGA, NCAM1)

The results of the in situ assay were in line with clinical parameters (Fig. 9A and C). Total PSA measurements of 6.75 ng/ml in PC-13 and 904.06 ng/ml in PC-16 were also reflected by CTC numbers and PSA expression levels determined by in situ analysis, with PSA positivity in 5 of 8 CTCs and a median overall expression of 2 PSA RCPs/cell (IQR 0–8) in PC-13, compared to PSA positivity in all 38 CTCs and a median expression of 10 PSA RCPs/cell (IQR 4–38) in PC-16. In PC-15, a patient with treatment emergent small-cell neuroendocrine PC, CTCs expressing neuroendocrine markers (SYP, CHGA, NCAM1) and DLL3 co-occurred with elevated blood levels of neuron-specific enolase (1451 ng/ml) and chromogranin A (2764 ng/ml). Similarly, in situ CTC analysis showed high agreement with the AdnaTest results (Fig. 9B and C). PSA, PSMA, AR-FL/AR, and AR-V7 were detected in patient PC-13 and PC-16 by both assays. In patient PC-15, the assays were concordant regarding the absence of PSA, AR-FL/AR, and AR-V7. Interestingly, low-level PSMA expression was detected by the AdnaTest but not by in situ analysis.

### CoDuCo analysis of patient-matched CTC and tissue samples

**Fig. 10:**
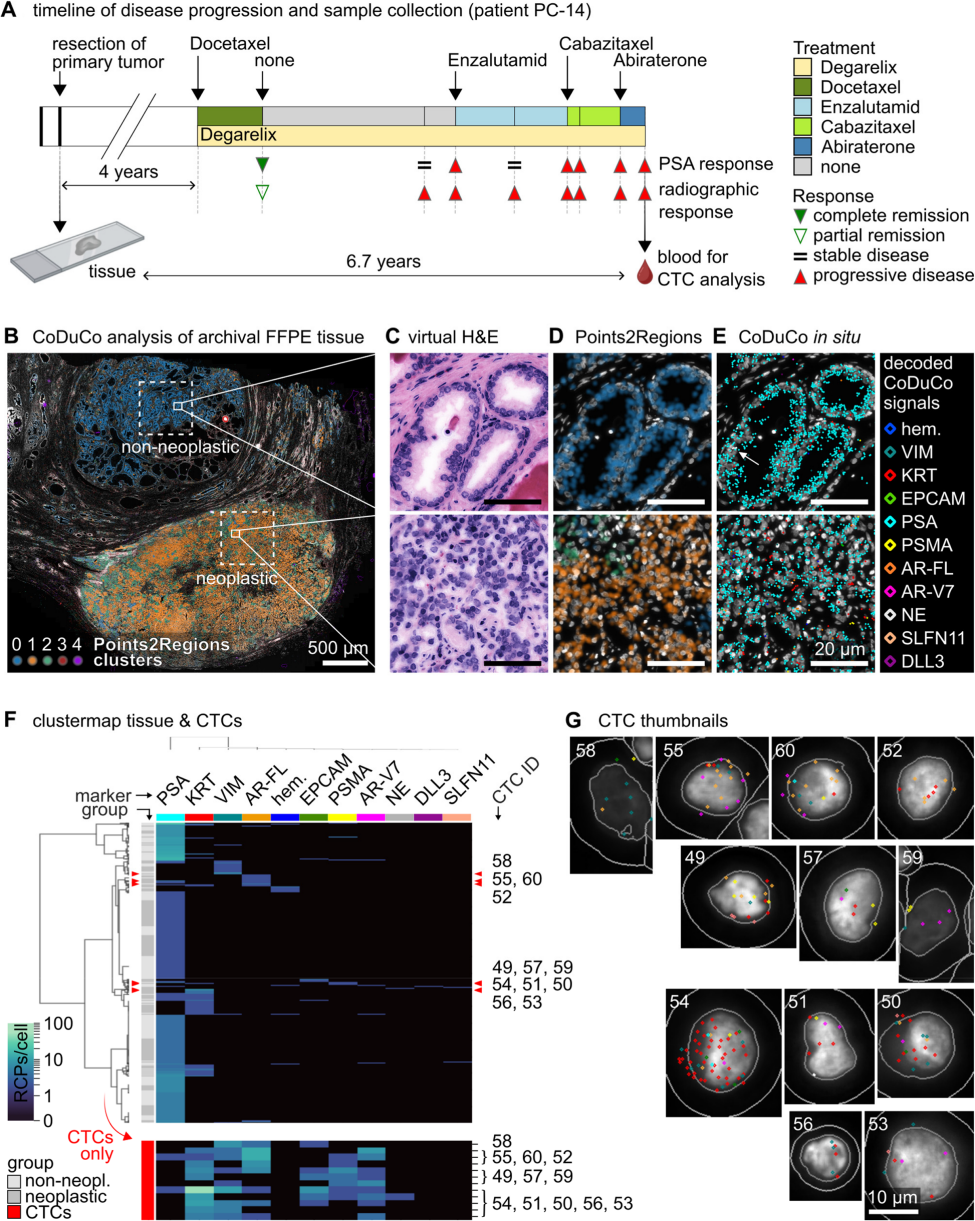
CoDuCo *in situ* analysis of matched tissue and CTC samples of patient PC-14. **A** Illustration of patient PC-14’s timeline of disease progression (treatments, PSA response, and radiographic response) and sample collection time points. Tissue sample and CTC sample are separated by 6.7 years of tumor evolution and multiple lines of treatment. **B**-**E** Results of CoDuCo analysis of archival FFPE tissue. **B** Overview of the tissue section with DAPI stained nuclei (white). The TissUUmaps plugin Points2Regions was used for coarse spatial clustering of CoDuCo signals. Color-coded clusters fit well to the histological tissue structure and indicate differences between non-neoplastic (blue) and neoplastic tissue (orange, green). **C**-**E** Selected non-neoplastic and neoplastic regions are shown in more detail, with **C** virtual H&E staining, **D** overlay of Points2Regions clusters, and **E** overlay of decoded *in situ* signals. The arrow highlights a neuroendocrine cell (NE marker, white diamond) in the non-neoplastic epithelium. **F** A subset of 10000 cells each were randomly chosen from selected regions (indicated by dashed rectangles in panel (**B**)) to perform hierarchical clustering of non-neoplastic tissue (light gray), neoplastic tissue (dark gray), and CTCs (red), based on single-cell expression profiles. The position of CTCs in the clustermap is highlighted by red arrowheads and corresponding CTC ID numbers. To better visualize the number of *in situ* signals/cell in CTCs, a separate heatmap is depicted at the bottom. **G** CTC thumbnails showing the DAPI image with CTC ID number, outlines of nuclei, cell borders, and decoded *in situ* signals as detected by CellProfiler analysis. The colors of decoded *in situ* signals is indicated for each marker on top of the clustermap (**F**) and the legend in panel **E**

Finally, we tested the applicability of the CoDuCo in situ assay on FFPE tissue. Archival FFPE tissue was available from the resection of the primary tumor of patient PC-14. A matched CTC sample from the same patient, collected 6.7 years later, after progression to metastatic disease and multiple lines of systemic treatment, was analyzed as well. The timeline of disease progression and sample collection is summarized in Fig. 10A. CoDuCo staining and decoding were successfully adapted to FFPE tissue, which enabled us to visualize expression patterns in non-neoplastic tissue, neoplastic tissue, and CTCs. We explored differences of expression patterns in the coarse spatial context using the TissUUmaps plugin Points2Regions. As depicted in Fig. 10B and D, distinct clusters were identified with a good fit to the histological structure of the sample with non-neoplastic epithelium (predominantly cluster 0), neoplastic tissue (cluster 1 and 2), stroma (cluster 3), and areas with decoding issues due to high autofluorescence or imaging artefacts (cluster 4). At single-cell resolution, hierarchical clustering showed no clear distinction between non-neoplastic and neoplastic tissue but instead revealed a more diverse set of cell populations, including rare neuroendocrine cells (Fig. 10E and F). CTC expression levels showed large differences to the tissue sample, with an overall decrease of PSA expression and increased expression of KRT, VIM, AR-FL, EPCAM, PSMA, and AR-V7 (Fig. 10F and G).

## Discussion

Our data show that CTCs can be identified and characterized by our novel CoDuCo in situ assay, which targets up to 11 markers in a multiplex fashion. Multiple predictive biomarkers are detected simultaneously, which previously was not feasible. Of utmost importance is the assay’s capability to detect neuroendocrine markers, as this remains challenging with conventional antibody staining procedures. Quantitative assessment of expression levels of CTCs is possible, and the machine learning classifier is a powerful tool to recognize CTCs. The assay provides single-cell resolution RNA expression data and images of cell morphology, which facilitates the identification of single CTCs and CTC clusters and strikingly reveals intrapatient heterogeneity.

### Neuroendocrine transdifferentiation is detectable in CTCs

The increased multiplex capacity of the CoDuCo in situ approach enabled us to visualize in total 11 markers, either as single transcripts (e.g. SLFN11, DLL3), or pooled markers (e.g. SYP, CHGA, NCAM1). The combination of markers increases the informative value which can be obtained from CTCs. Simultaneous assessment of 11 targets is difficult to accomplish with conventional antibody staining or would require sophisticated and expensive targeted proteomics mass cytometry techniques [63]. Using our CoDuCo assay, we could clearly identify CTCs, but more importantly, we could investigate multiple resistance markers on a single-cell level. Of high clinical importance is the exploration of neuroendocrine transdifferentiation, which is an AR-independent resistance mechanism in CRPC. While the incidence of neuroendocrine PC is rising, its early detection and treatment remain difficult and improvements are urgently needed [8, 64, 65]. Thanks to the CoDuCo in situ assay's improved multiplexing capability, we were able to add markers relevant for neuroendocrine PC to our panel. We visualized SYP, CHGA, and NCAM1 transcripts as pooled neuroendocrine markers to identify CTCs with neuroendocrine features, as well as DLL3 and SLFN11 as potential predictive markers. Indeed, we successfully identified neuroendocrine CTCs in a patient with diagnosed treatment-emergent neuroendocrine PC (PC-15) in our proof of principle study. A large patient cohort would be needed to determine whether in situ CTC analysis can reveal neuroendocrine transdifferentiation earlier or with higher specificity than the serum markers currently used in the clinic. However, a clear advantage of our approach is the parallel detection of DLL3 and SLFN11 expression in neuroendocrine CTCs. SLFN11 expression is a potential positive predictive biomarker for platinum-based chemotherapy and PARP inhibitors, and thus relevant for neuroendocrine PC [27, 66, 67]. In contrast, DLL3 has been associated with resistance against platinum-based chemotherapy [68, 69]. Notably, DLL3 itself is a potential therapeutic target, which is currently investigated in several preclinical and clinical studies [16, 70]. So far, only a limited number of studies investigated and confirmed the detection of DLL3 expression in CTCs of CRPC and small cell lung cancer patients [70–72]. Although DLL3 expression in CTCs is representative of expression in matched metastatic tissue biopsies [70], conflicting data exists regarding DLL3’s predictive value in the context of DLL3-targeted therapy and further investigation is needed [73]. In the case presented here, we detected three neuroendocrine CTCs. SLFN11 and DLL3 were detected in two of three CTCs, respectively. Coexpression of SLFN11 and DLL3 was observed in one CTC, while the remaining CTCs expressed either SLFN11 or DLL3. An interesting finding is that neuroendocrine CTCs still expressed keratin, despite having no prostate-specific transcripts such as PSA, PSMA or AR-FL. This might be important for other CTC-based assays that use keratin staining as inclusion marker, implying they can still identify CTCs in neuroendocrine PC patients. Although these data of a single case merely confirm the capability to detect neuroendocrine markers in CTCs, the findings suggest that this assay may bring novel insights regarding the treatment options and resistance mechanisms in neuroendocrine PC. Notably, the mechanisms driving neuroendocrine transdifferentiation are complex, involving genomic, epigenetic, transcriptional, and post-translational changes [8]. Our approach utilizes commonly employed markers to investigate neuroendocrine-differentiated cells; however, the clinical relevance of specific markers, such as SLFN11 and DLL3 in CRPC, requires further investigation.

### PSMA expression is detectable in CTCs

Similarly, the increased multiplex capacity of the CoDuCo approach enabled us to include PSMA as additional prostate-specific marker. Novel PSMA-targeting drugs, such as the radioligand agent Lutetium-177 PSMA-617 (trade name: Pluvicto) [18], are now available, and others are under investigation [17, 74]. Predictive biomarkers are urgently needed, as the expression of PSMA is highly heterogeneous and dynamic [75]. PSMA upregulation by ADT, by inhibition of the PI3K/Akt/mTOR pathway, and by cell stress through DNA damaging treatments or DNA damage response inhibitors have been described, which implies potential benefits of combination treatments [76]. Thus, visualizing PSMA expression in CTCs might not only prove useful for patient stratification, but may, in the context of a comprehensive multi-analyte liquid biopsy approach, lead to a better understanding of PSMA regulation and potential implications for combination treatments [77]. In the data presented here, we detected PSMA-expressing CTCs in two patients but none in the patient with treatment-emergent neuroendocrine PC, where we detected three CTCs expressing KRT and neuroendocrine markers, but no prostate-specific markers. These findings are in line with published data demonstrating decreased or absent PSMA expression in AR-negative PC [76, 78]. In contrast to the CoDuCo in situ results, PSMA expression was detected by the AdnaTest assay in all samples. The discordance between the two assays may point to differences in sensitivity and/or specificity and should be investigated in a larger cohort of PC patients and healthy controls. Importantly, in high risk/advanced PC patients the current gold standard technique for PSMA detection is a whole-body imaging with positron emission tomography (PET) using small amounts of radioactive tracers, such as 68Ga-PSMA-11 [79]. Utilizing a liquid biopsy assay as an alternative to PET imaging with radioactive tracers would be highly beneficial for patients and healthcare providers. PSMA expressing CTCs might be used for patient stratification, monitoring the efficacy of Lutetium-177 PSMA-617 radioligand treatment, and identifying potential resistance mechanisms to this novel treatment option.

### AR-FL, AR-V7, and PSA are detectable and align with clinical serum markers

We designed our CoDuCo in situ assay to visualize the expression of AR-FL, AR-V7, and PSA, among others. In comparison to our previous study [38], the detection of AR-FL and PSA was optimized by increasing the number of PLPs per transcript from a single PLP to seven and six PLPs, respectively. As inhibition of the AR pathway is the mainstay of systemic PC treatment and AR-V7 is a well described resistance marker in up to 25% of CRPC patients, assessing patients’ AR status over the course of the treatment is highly relevant [80]. PSA is an AR-regulated gene, meaning that its expression can be interpreted as a measurement for AR-pathway activity when monitoring the response to AR-targeted treatments [34]. Furthermore, serum PSA is the most popular biomarker in PC and is routinely monitored in the clinical setting. We found high concordance between serum PSA levels and CoDuCo in situ derived expression levels of PSA-positive CTCs. This concordance supports our CoDuCo in situ results with actual clinical data and strengthens our assay.

### Single-cell resolution reveals tumor heterogeneity in CTCs and CTC clusters

The CoDuCo in situ assay delivers single-cell resolution RNA expression data and images of cell morphology, thereby enabling the identification of single CTCs and CTC clusters, and the exploration of tumor heterogeneity. We observed intrapatient CTC heterogeneity in two patients, namely, PC-13 and PC-16. In PC-16 we found substantial heterogeneity of KRT, PSA, AR-FL, and AR-V7 expression levels between CTCs. However, the expression levels of all these markers followed a similar gradient, from low expression of all markers to high expression of all markers, suggesting that the heterogeneity may have been caused by transcriptional activity overall, rather than multiple clones preexisting in the tumor mass (cellular plasticity). In contrast, PC-13 did not follow this pattern. Instead, there were at least two CTC cell populations, one with remarkably high coexpression of KRT and VIM in the absence of AR-V7, and one with more prominent expression of prostate-specific markers, especially PSA and AR-V7. This might indicate clonal heterogeneity of the tumor mass as described previously [81]. Overall, CTC heterogeneity has been described as predictive biomarker in metastatic CRPC [82], highlighting the importance of single-cell-based approaches like our CoDuCo in situ assay, which can visualize and quantify heterogeneity among CTCs. Besides heterogeneity on transcriptional level, a potential extension of our CoDuCo assay is the simultaneous detection of RNA gene expression and genetic point mutations as previously described [83–85]. This would enable investigation of heterogeneity on genomic and transcriptomic level. In PC-16, we detected CTC clusters with and without associated PBMCs or in situ-negative cells. The CTC clusters differed both in their expression patterns as well as their size, expressing KRT, PSA, AR-FL, and in some cases AR-V7, but no VIM, and the largest cluster contained nine CTCs. The detection and analysis of CTC clusters is highly relevant, as they are associated with high metastatic potential and poor outcomes [86–88] and drugs that target and disaggregate CTC clusters are under investigation in preclinical and clinical (NCT03928210) studies [89, 90].

### Single-cell and spatial tissue analysis

We investigated the applicability of CoDuCo staining on archival FFPE tissue from a matched patient sample, comparing it with CTC CoDuCo data. Our findings demonstrate that CoDuCo can be successfully applied to FFPE tissue sections, facilitating spatial analysis at the single-cell level. Differential gene expression analysis between CoDuCo CTCs and archival CoDuCo tissue revealed significant differences, consistent with the hypothesis that tumors evolve under therapeutic pressure through the expansion of resistant subclones [91]. In this particular patient sample, the pronounced differences can be attributed to the 6.7-year interval and multiple lines of therapy between tissue resection and liquid biopsy collection. However, a common feature among all CTCs was the downregulation of PSA expression relative to the archival tissue. Notably, AR-FL and AR-V7 transcripts were detected in the CTCs, indicating the presence of AR-dependent resistance mechanisms in this patient [82]. Our approach to investigate the spatial arrangement of matched tissue and CTC expression profiles with CoDuCo staining may yield new insights into CTC shedding mechanisms, when applied to time-matched tissue and CTC samples.

### Image analysis

A challenge for the CoDuCo assay is the complexity of image analysis. In our previous study, a four-channel fluorescence microscope limited our conventional in situ PLP hybridization approach to detecting only three markers, but image analysis was comparably simple [38]. In our novel CoDuCo in situ assay, we used a seven-channel fluorescence microscope and dual-color combinations to detect in situ signals. In theory, this combinatorial approach can distinguish in situ signals with up to 15 unique color codes [92]. However, with CoDuCo staining, a highly optimized, crosstalk-free filter setup is required to ensure specificity. As we observed some bleed-through of Cy5-labelled signals into the TexasRed channel, we used TexasRed only for a single color-code instead of five, ensuring high specificity.

To ease image analysis and sample evaluation, we developed a semi-automated image analysis pipeline using Python libraries and CellProfiler and established machine learning-assisted classification of cells. Using this pipeline, we decoded colocalized in situ signals and assigned them to cells that we detected based on their DAPI-stained nuclei. Although the automation of image analysis ensured the feasibility of the CoDuCo in situ analysis of CTCs, we still encountered technical limitations and therefore further optimizations will be indispensable. These limitations included wide variations of the intensity values of DAPI staining, in situ signals, but also background autofluorescence of patient samples. Although our CellProfiler pipeline used automated thresholding algorithms to detect nuclei and in situ signals, some parameters needed to be checked and adjusted for each individual sample to ensure optimal segmentation results. In addition to that, we stripped the in situ signals from the samples and created a background scan to be subtracted from the original images to increase the signal to background ratio [45]. Both the manual adjustments and the background scan are time-consuming and therefore, optimizations would be worthwhile. The use of machine learning and deep learning-based tools for deconvolution, spot enhancement, and image segmentation (e.g. Cellpose, StarDist, ilastik, DeepSpot, Deconwolf) might improve the detection of nuclei and in situ signals and speed up the analysis workflow [93–97]. Moreover, as our CoDuCo in situ assay involved no cytoplasm or membrane staining, we enlarged detected nuclei, based on their size, by a specified number of pixels to capture the whole cells. Implementing membrane or cytoplasmic staining, such as cell painting tools, to guide the detection of cell borders, would be another potential improvement to the image analysis workflow [98, 99]. The entire CoDuCo workflow for a single blood sample—including sample processing, in situ staining, scanning, and image analysis—takes approximately 3 to 4 days. Specifically, CTC enrichment requires 2 h, in situ staining takes 2 days, imaging takes 2 to 4 h, and image analysis with machine learning classification takes 1 to 5 h computing time. Manual image evaluation of dual-positive in situ signals would be extremely time-consuming, and challenging to scale for future clinical applications.

### Machine learning-based classification

To train a classifier for CTC detection by supervised machine learning, we first had to create a ground-truth dataset. We used blood samples of healthy controls with and without spiked-in VCaP and PC-3 cells, enriched them for CTCs using CytoGen’s Smart Biopsy Cell Isolator, and performed CoDuCo in situ analysis. We manually annotated the dataset and trained a random forest classifier using the CellProfiler Analyst software. When tested on three samples of PC patients, the classifier reached a recall of 0.76 and specificity of 0.99, meaning that 76% of CTCs and 99% of non-CTCs were correctly recognized, and a precision of 0.17, meaning that 17% of predicted CTCs were true CTCs.

The observed discrepancy of performance metrics between the healthy controls’ spike-in samples and the patient samples indicated that training and test dataset were not representative for patient samples. This finding was expected as there is a large difference between cultured cancer cell lines and patient CTCs [100]. The importance of a representative training dataset to correctly identify CTCs was also noted by others, who used deep-learning convolutional neural networks to classify CTCs, either via deep-learning or by operator reviewed CTCs [101]. Since the identification of CTCs can be difficult even for experts, the creation of a ground truth dataset is very challenging. Therefore, including additional samples of healthy controls is of particular importance to minimize false-positive CTC calls and improve classifier performance overall [101]. Also, identification of CTCs might be improved by including morphological features, so that classification is based on molecular (mRNA in situ signals) and cellular (e.g. nucleus shape) features. Eventually re-training of the classifier is needed, using a more representative dataset [102]. The most suitable ground truth dataset for classifier training will be a collection of several hundred CoDuCo in situ hybridized samples from healthy controls and PC patients with a large number of expert-reviewed patient CTCs, reflecting the variety of phenotypes and heterogeneity of CTCs. The creation of such a dataset will be necessary and will ultimately serve as the foundation for a better classifier. Nevertheless, the benefit of the current classifier, despite its limitations, becomes evident when considering absolute numbers. In the three patient samples used to evaluate classifier performance, a total of 17,756 cells were detected, of which 49 were CTCs. The classifier labeled 214 cells as CTCs, of which 37 were true CTCs. Even with this low precision of 0.17, the classifier still represents a major improvement over manual classification, considering that only 214 instead of 17,756 cells need to be manually inspected, leading to a dramatic reduction in hands-on time. At the same time, the large number of cells also highlights that manually annotating a more extensive training and test dataset with hundreds of patient samples will be a time-intensive task, albeit one that will be essential for future advancements.

## Conclusion

We demonstrated the feasibility of a novel CoDuCo in situ approach which identifies CTCs with high sensitivity and specificity. CoDuCo staining increases the multiplex capacity of this assay, allowing us to visualize a more comprehensive panel of transcripts, including neuroendocrine, epithelial, prostate-specific, mesenchymal, and hematopoietic markers in tissue and cells. The transcripts were selected to inform about diverse resistance mechanisms (AR-V7 expression, neuroendocrine transdifferentiation), druggable targets and predictive markers (PSMA, DLL3, SLFN11), and cancer-related processes such as epithelial mesenchymal transition (VIM, KRT). To ensure practical applicability, we implemented semi-automated image analysis combined with machine learning-assisted CTC classification. A unique advantage of the CoDuCo in situ assay is the combination of high multiplex capacity and microscopy-based single-cell analysis, which is instrumental to simultaneously identify and characterize CTCs, detect CTC clusters, and visualize CTC heterogeneity. Ultimately, the assay is a promising tool for tracking the evolving molecular alterations linked to drug response and resistance in PC and enables the analysis of matched tissue- and liquid biopsy samples.

## Data Availability

The datasets used and/or analyzed during the current study are available from the corresponding author on reasonable request. The Python scripts and CellProfiler pipelines developed for the CoDuCo in situ CTC analysis workflow are deposited in a public repository and can be accessed openly: https://doi.org/10.5281/zenodo.11125572.

## Abbreviations

ADT: Androgen deprivation therapy
AR: Androgen receptor
AR-FL: Androgen receptor full length
AR-V: Androgen receptor splice variant
AR-V7: Androgen receptor splice variant 7
CD45: Cluster of differentiation 45
CHGA: Chromogranin A
CoDuCo: Combinatorial dual-color
CRPC: Castration resistant prostate cancer
csv: Comma separated value
CTC: Circulating tumor cell
ctDNA: Circulating tumor DNA
Cy3, Cy5: Cyanin-3, Cyanin-5
DAPI: 4′,6-Diamidino-2-phenylindole
DEPC: Diethylpyrocarbonate
DLL3: Delta-like-protein 3, delta-like canonical Notch ligand 3
EDTA: Ethylenediaminetetraacetic acid
EPCAM, EpCAM: Epithelial cell adhesion molecule
FBS: Fetal bovine serum
FCGR3: Fc gamma receptor III
FFPE: Formalin-fixed, paraffin-embedded
FOLH1: Folate hydrolase 1
HCl: Hydrochloric acid
HDM chip: High density microporous chip
IQR: Interquartile range
ITGAM: Integrin subunit alpha M
ITGB2: Integrin subunit beta 2
KCl: Potassium chloride
KLK3: Kallikrein related peptidase 3
KRT8, KRT18, KRT19: Keratin 8, 18, and 19
LDH: Lactate dehydrogenase
NCAM1: Neural cell adhesion molecule 1
NE: Neuroendocrine
NEPC: Neuroendocrine prostate cancer
NSE: Neuron specific enolase
PARP: Poly ADP ribose polymerase
PBMC: Peripheral blood mononuclear cell
PBS: Phosphate buffered saline
PC: Prostate cancer
PLP: Padlock probe
PSA: Prostate specific antigen
PSMA: Prostate specific membrane antigen
PTPRC: Protein tyrosine phosphatase receptor type C
px: Pixel
RCP: Rolling circle product
ROI: Region of interest
SLFN11: Schlafen family member 11
SSC: Saline sodium citrate
SYP: Synaptophysin
TR: TexasRed
VIM: Vimentin

## References

[1] Ferlay J, Colombet M, Soerjomataram I, Parkin DM, Piñeros M, Znaor A, et al. Cancer statistics for the year 2020: An overview. Int J Cancer. 2021;149:778–89. 10.1002/ijc.33588 33818764

[2] Sung H, Ferlay J, Siegel RL, Laversanne M, Soerjomataram I, Jemal A, et al. Global Cancer Statistics 2020: GLOBOCAN Estimates of Incidence and Mortality Worldwide for 36 Cancers in 185 Countries. CA: A Cancer Journal for Clinicians. 2021;71:209–49. 10.3322/caac.21660 33538338

[3] Gann PH. Risk Factors for Prostate Cancer. Rev Urol. 2002;4:S3-10. 16986064 PMC1476014

[4] Siegel RL, Miller KD, Fuchs HE, Jemal A. Cancer statistics, 2022. CA: A Cancer Journal for Clinicians. 2022;72:7–33. 10.3322/caac.21708 35020204

[5] James ND, Tannock I, N’Dow J, Feng F, Gillessen S, Ali SA, et al. The Lancet Commission on prostate cancer: planning for the surge in cases. The Lancet [Internet]. 2024 [cited 2024 Apr 23]; Available from: https://www.sciencedirect.com/science/article/pii/S0140673624006512. 10.1016/S0140-6736(24)00651-2 PMC7617369 38583453

[6] Carreira S, Romanel A, Goodall J, Grist E, Ferraldeschi R, Miranda S, et al. Tumor clone dynamics in lethal prostate cancer. Sci Transl Med. 2014;6:254ra12 10.1126/scitranslmed.3009448 PMC4422178 25232177

[7] Prekovic S, Van den Broeck T, Moris L, Smeets E, Claessens F, Joniau S, et al. Treatment-induced changes in the androgen receptor axis: Liquid biopsies as diagnostic/prognostic tools for prostate cancer. Mol Cell Endocrinol. 2018;462:56–63. 28882555 10.1016/j.mce.2017.08.020

[8] Merkens L, Sailer V, Lessel D, Janzen E, Greimeier S, Kirfel J, et al. Aggressive variants of prostate cancer: underlying mechanisms of neuroendocrine transdifferentiation. J Exp Clin Cancer Res. 2022;41:46. 35109899 10.1186/s13046-022-02255-y PMC8808994

[9] Antonarakis ES, Lu C, Wang H, Luber B, Nakazawa M, Roeser JC, et al. AR-V7 and Resistance to Enzalutamide and Abiraterone in Prostate Cancer. N Engl J Med. 2014;371:1028–38. 25184630 10.1056/NEJMoa1315815 PMC4201502

[10] Ulz P, Belic J, Graf R, Auer M, Lafer I, Fischereder K, et al. Whole-genome plasma sequencing reveals focal amplifications as a driving force in metastatic prostate cancer. Nat Commun [Internet]. 2016 [cited 2019 Mar 30];7. Available from: https://www.ncbi.nlm.nih.gov/pmc/articles/PMC4917969/10.1038/ncomms12008 PMC4917969 27328849

[11] Robinson D, Van Allen EM, Wu Y-M, Schultz N, Lonigro RJ, Mosquera J-M, et al. Integrative Clinical Genomics of Advanced Prostate Cancer. Cell. 2015;161:1215–28. 26000489 10.1016/j.cell.2015.05.001 PMC4484602

[12] Kohli M, Ho Y, Hillman DW, Van Etten JL, Henzler C, Yang R, et al. Androgen Receptor Variant AR-V9 Is Coexpressed with AR-V7 in Prostate Cancer Metastases and Predicts Abiraterone Resistance. Clin Cancer Res. 2017;23:4704–15. 28473535 10.1158/1078-0432.CCR-17-0017 PMC5644285

[13] Antunac K, Beketić-Orešković L. HOW TO OPTIMALLY SEQUENCE AVAILABLE THERAPY LINES IN ADVANCED PROSTATE CANCER. Acta Clin Croat. 2022;61:32–44. 36938556 10.20471/acc.2022.61.s3.5 PMC10022401

[14] Shore ND, Morgans AK, El-Haddad G, Srinivas S, Abramowitz M. Addressing Challenges and Controversies in the Management of Prostate Cancer with Multidisciplinary Teams. Target Oncol. 2022;17:709–25. 36399218 10.1007/s11523-022-00925-7 PMC9672595

[15] Antonarakis ES, Lu C, Luber B, Wang H, Chen Y, Nakazawa M, et al. Androgen Receptor Splice Variant 7 and Efficacy of Taxane Chemotherapy in Patients With Metastatic Castration-Resistant Prostate Cancer. JAMA Oncol. 2015;1:582. 26181238 10.1001/jamaoncol.2015.1341 PMC4537351

[16] Beltran H, Demichelis F. Therapy considerations in neuroendocrine prostate cancer: what next? Endocr Relat Cancer. 2021;28:T67-78. 34111024 10.1530/ERC-21-0140 PMC8289743

[17] Cornford P, van den Bergh RCN, Briers E, Van den Broeck T, Cumberbatch MG, De Santis M, et al. EAU-EANM-ESTRO-ESUR-SIOG Guidelines on Prostate Cancer. Part II—2020 Update: Treatment of Relapsing and Metastatic Prostate Cancer. European Urology. 2020;79:263–82. 10.1016/j.eururo.2020.09.046 33039206

[18] Sartor O, de Bono J, Chi KN, Fizazi K, Herrmann K, Rahbar K, et al. Lutetium-177–PSMA-617 for Metastatic Castration-Resistant Prostate Cancer. N Engl J Med. 2021;385:1091–103. 34161051 10.1056/NEJMoa2107322 PMC8446332

[19] Pantel K, Alix-Panabières C. Circulating tumour cells in cancer patients: challenges and perspectives. Trends Mol Med. 2010;16:398–406. 20667783 10.1016/j.molmed.2010.07.001

[20] Belic J, Graf R, Bauernhofer T, Cherkas Y, Ulz P, Waldispuehl-Geigl J, et al. Genomic alterations in plasma DNA from patients with metastasized prostate cancer receiving abiraterone or enzalutamide: Prostate cancer and plasma DNA analyses. Int J Cancer. 2018;143:1236–48. 29574703 10.1002/ijc.31397 PMC6099279

[21] Alix-Panabières C, Pantel K. Liquid biopsy: from discovery to clinical implementation. Mol Oncol. 2021;15:1617–21. 34075709 10.1002/1878-0261.12997 PMC8169443

[22] Tukachinsky H, Madison RW, Chung JH, Gjoerup O, Severson EA, Dennis L, et al. Genomic analysis of circulating tumor DNA in 3,334 patients with advanced prostate cancer identifies targetable BRCA alterations and AR resistance mechanisms. Clin Cancer Res. 2021;clincanres.CCR-20–4805-E.2020. 10.1158/1078-0432.CCR-20-4805 PMC9295199 33558422

[23] Sharp A, Welti JC, Lambros MBK, Dolling D, Rodrigues DN, Pope L, et al. Clinical Utility of Circulating Tumour Cell Androgen Receptor Splice Variant-7 Status in Metastatic Castration-resistant Prostate Cancer. Eur Urol. 2019;76(5):676–85. ;S0302283819302854. 10.1016/j.eururo.2019.04.006 31036442

[24] Scher HI, Lu D, Schreiber NA, Louw J, Graf RP, Vargas HA, et al. Association of AR-V7 on Circulating Tumor Cells as a Treatment-Specific Biomarker With Outcomes and Survival in Castration-Resistant Prostate Cancer. JAMA Oncol. 2016;2:1441. 27262168 10.1001/jamaoncol.2016.1828 PMC5206761

[25] Kanayama M, Lu C, Luo J, Antonarakis ES. AR Splicing Variants and Resistance to AR Targeting Agents. Cancers (Basel). 2021;13:2563. 34071114 10.3390/cancers13112563 PMC8197115

[26] Gupta S, Halabi S, Yang Q, Roy A, Tubbs A, Gore Y, et al. PSMA-positive Circulating Tumor Cell Detection and Outcomes with Abiraterone or Enzalutamide Treatment in Men with Metastatic Castrate-resistant Prostate Cancer. Clin Cancer Res. 2023;29:1929–37. 36897758 10.1158/1078-0432.CCR-22-3233 PMC10192124

[27] Scher HI, Fernandez L, Cunningham K, Elphick N, Barnett E, Lee J, et al. Schlafen 11 (SLFN11), a putative predictive biomarker of platinum/PARPi response, is frequently detected on circulating tumor cells (CTCs) in advanced prostate cancer. JCO. 2021;39:e17039–e17039.

[28] Gorges TM, Riethdorf S, von Ahsen O, Nastał YP, Röck K, Boede M, et al. Heterogeneous PSMA expression on circulating tumor cells: a potential basis for stratification and monitoring of PSMA-directed therapies in prostate cancer. Oncotarget. 2016;7:34930–41. 27145459 10.18632/oncotarget.9004 PMC5085200

[29] Autio KA, Dreicer R, Anderson J, Garcia JA, Alva A, Hart LL, et al. Safety and Efficacy of BIND-014, a Docetaxel Nanoparticle Targeting Prostate-Specific Membrane Antigen for Patients With Metastatic Castration-Resistant Prostate Cancer: A Phase 2 Clinical Trial. JAMA Oncol. 2018;4:1344–51. 29978216 10.1001/jamaoncol.2018.2168 PMC6233779

[30] Pantel K, Hille C, Scher HI. Circulating Tumor Cells in Prostate Cancer: From Discovery to Clinical Utility. Clin Chem. 2019;65:87–99. 30602476 10.1373/clinchem.2018.287102

[31] Kessel K, Seifert R, Weckesser M, Roll W, Humberg V, Schlack K, et al. Molecular analysis of circulating tumor cells of metastatic castration-resistant Prostate Cancer Patients receiving 177Lu-PSMA-617 Radioligand Therapy. Theranostics. 2020;10:7645–55. 32685010 10.7150/thno.44556 PMC7359074

[32] Zavridou M, Smilkou S, Tserpeli V, Sfika A, Bournakis E, Strati A, et al. Development and Analytical Validation of a 6-Plex Reverse Transcription Droplet Digital PCR Assay for the Absolute Quantification of Prostate Cancer Biomarkers in Circulating Tumor Cells of Patients with Metastatic Castration-Resistant Prostate Cancer. Clin Chem. 2022;68:1323–35. 36093578 10.1093/clinchem/hvac125

[33] Beltran H, Jendrisak A, Landers M, Mosquera JM, Kossai M, Louw J, et al. The Initial Detection and Partial Characterization of Circulating Tumor Cells in Neuroendocrine Prostate Cancer. Clin Cancer Res. 2016;22:1510–9. 26671992 10.1158/1078-0432.CCR-15-0137 PMC4990782

[34] Sperger JM, Emamekhoo H, McKay RR, Stahlfeld CN, Singh A, Chen XE, et al. Prospective Evaluation of Clinical Outcomes Using a Multiplex Liquid Biopsy Targeting Diverse Resistance Mechanisms in Metastatic Prostate Cancer. JCO. 2021;39:2926–37. 10.1200/JCO.21.00169 PMC8425833 34197212

[35] Zhao SG, Sperger JM, Schehr JL, McKay RR, Emamekhoo H, Singh A, et al. A clinical-grade liquid biomarker detects neuroendocrine differentiation in prostate cancer. J Clin Invest. 2022;132: e161858. 36317634 10.1172/JCI161858 PMC9621140

[36] Welsch E, Bonstingl L, Holzer B, Schuster E, Weiß E, Zaharie A-T, et al. Multi-marker analysis of circulating tumor cells in localized intermediate/high-risk and metastatic prostate cancer. Clin Exp Metastasis. 2024. Available from: 10.1007/s10585-024-10313-2. 10.1007/s10585-024-10313-2 PMC11607046 39305397

[37] De Sarkar N, Patton RD, Doebley A-L, Hanratty B, Adil M, Kreitzman AJ, et al. Nucleosome Patterns in Circulating Tumor DNA Reveal Transcriptional Regulation of Advanced Prostate Cancer Phenotypes. Cancer Discov. 2023;13:632–53. 36399432 10.1158/2159-8290.CD-22-0692 PMC9976992

[38] Hofmann L, Sallinger K, Haudum C, Smolle M, Heitzer E, Moser T, et al. A Multi-Analyte Approach for Improved Sensitivity of Liquid Biopsies in Prostate Cancer. Cancers. 2020;12:2247. 32796730 10.3390/cancers12082247 PMC7465186

[39] Bonstingl L, Skofler C, Ulz C, Zinnegger M, Sallinger K, Schönberger J, et al. Clinical Application of ISO and CEN/TS Standards for Liquid Biopsies—Information Everybody Wants but Nobody Wants to Pay For. Clinical Chemistry. 2024;hvae079. 10.1093/clinchem/hvae079 38958115

[40] Hofmann L, Kroneis T, El-Heliebi A. Using In Situ Padlock Probe Technology to Detect mRNA Splice Variants in Tumor Cells. In: Nielsen BS, Jones J, editors. In Situ Hybridization Protocols [Internet]. New York, NY: Springer US; 2020 [cited 2020 May 15]. p. 361–78. Available from: 10.1007/978-1-0716-0623-0_23 10.1007/978-1-0716-0623-0_23 32394394

[41] Lee SJ, Lee CH, Choi SH, Ahn SH, Son BH, Lee JW, et al. Evaluation of a novel approach to circulating tumor cell isolation for cancer gene panel analysis in patients with breast cancer. Oncol Lett. 2017;13:3025–31. 28521409 10.3892/ol.2017.5807 PMC5431305

[42] Larsson C, Grundberg I, Söderberg O, Nilsson M. In situ detection and genotyping of individual mRNA molecules. Nat Methods. 2010;7:395–7. 20383134 10.1038/nmeth.1448

[43] Gyllborg D, Langseth CM, Qian X, Choi E, Salas SM, Hilscher MM, et al. Hybridization-based in situ sequencing (HybISS) for spatially resolved transcriptomics in human and mouse brain tissue. Nucleic Acids Res. 2020;48: e112. 32990747 10.1093/nar/gkaa792 PMC7641728

[44] Weibrecht I, Lundin E, Kiflemariam S, Mignardi M, Grundberg I, Larsson C, et al. In situ detection of individual mRNA molecules and protein complexes or post-translational modifications using padlock probes combined with the in situ proximity ligation assay. Nat Protoc. 2013;8:355–72. 23348363 10.1038/nprot.2013.006

[45] Sallinger K, Gruber M, Müller C-T, Bonstingl L, Pritz E, Pankratz K, et al. Spatial tumour gene signature discriminates neoplastic from non-neoplastic compartments in colon cancer: unravelling predictive biomarkers for relapse. J Transl Med. 2023;21:528. 37543577 10.1186/s12967-023-04384-0 PMC10403907

[46] Stirling DR, Swain-Bowden MJ, Lucas AM, Carpenter AE, Cimini BA, Goodman A. Cell Profiler 4: improvements in speed, utility and usability. BMC Bioinformatics. 2021;22:433. 34507520 10.1186/s12859-021-04344-9 PMC8431850

[47] Thévenaz P, Ruttimann UE, Unser M. A pyramid approach to subpixel registration based on intensity. IEEE Trans Image Process. 1998;7:27–41. 18267377 10.1109/83.650848

[48] Stirling DR, Carpenter AE, Cimini BA. CellProfiler Analyst 3.0: accessible data exploration and machine learning for image analysis. Bioinformatics. 2021;37:3992–4. 10.1093/bioinformatics/btab634 PMC10186093 34478488

[49] Pielawski N, Andersson A, Avenel C, Behanova A, Chelebian E, Klemm A, et al. TissUUmaps 3: Improvements in interactive visualization, exploration, and quality assessment of large-scale spatial omics data. Heliyon. 2023;9: e15306. 37131430 10.1016/j.heliyon.2023.e15306 PMC10149187

[50] Andersson A, Behanova A, Avenel C, Windhager J, Malmberg F, Wählby C. Points2Regions: Fast, interactive clustering of imaging-based spatial transcriptomics data. Cytometry Part A [Internet]. [cited 2024 Aug 13];n/a. Available from: https://onlinelibrary.wiley.com/doi/abs/10.1002/cyto.a.24884. 10.1002/cyto.a.24884 38958502

[51] Waskom ML. seaborn: statistical data visualization. Journal of Open Source Software. 2021;6:3021.

[52] Van Rossum G, Drake FL. Python 3 Reference Manual. Scotts Valley, CA: CreateSpace; 2009.

[53] McKinney W, others. Data structures for statistical computing in python. Proceedings of the 9th Python in Science Conference. Austin, TX; 2010. p. 51–6.

[54] Harris CR, Millman KJ, van der Walt SJ, Gommers R, Virtanen P, Cournapeau D, et al. Array programming with NumPy. Nature. 2020;585:357–62. 32939066 10.1038/s41586-020-2649-2 PMC7759461

[55] Seabold S, Perktold J. statsmodels: Econometric and statistical modeling with python. 9th Python in Science Conference. 2010.

[56] Virtanen P, Gommers R, Oliphant TE, Haberland M, Reddy T, Cournapeau D, et al. SciPy 1.0: Fundamental Algorithms for Scientific Computing in Python. Nature Methods. 2020;17:261–72. 10.1038/s41592-019-0686-2 PMC7056644 32015543

[57] Terpilowski MA. scikit-posthocs: Pairwise multiple comparison tests in Python. Journal of Open Source Software. 2019;4:1169.

[58] Pedregosa F, Varoquaux G, Gramfort A, Michel V, Thirion B, Grisel O, et al. Scikit-learn: Machine learning in Python. J Mach Learn Res. 2011;12:2825–30.

[59] Chicco D, Jurman G. The advantages of the Matthews correlation coefficient (MCC) over F1 score and accuracy in binary classification evaluation. BMC Genomics. 2020;21:6. 31898477 10.1186/s12864-019-6413-7 PMC6941312

[60] Chicco D, Jurman G. The Matthews correlation coefficient (MCC) should replace the ROC AUC as the standard metric for assessing binary classification. BioData Min. 2023;16:4. 36800973 10.1186/s13040-023-00322-4 PMC9938573

[61] Hunter JD. Matplotlib: A 2D graphics environment. Computing in science & engineering. 2007;9:90–5.

[62] Kluyver T, Ragan-Kelley B, P&#233, Rez F, Granger B, Bussonnier M, et al. Jupyter Notebooks – a publishing format for reproducible computational workflows. Positioning and Power in Academic Publishing: Players, Agents and Agendas [Internet]. IOS Press; 2016 [cited 2023 Jun 18]. p. 87–90. Available from: https://ebooks.iospress.nl/doi/10.3233/978-1-61499-649-1-87.

[63] Chai S, Ruiz-Velasco C, Naghdloo A, Pore M, Singh M, Matsumoto N, et al. Identification of epithelial and mesenchymal circulating tumor cells in clonal lineage of an aggressive prostate cancer case. npj Precis Onc. 2022;6:1–11. 10.1038/s41698-022-00289-1 PMC9213535 35729213

[64] Patel GK, Chugh N, Tripathi M. Neuroendocrine Differentiation of Prostate Cancer-An Intriguing Example of Tumor Evolution at Play. Cancers (Basel). 2019;11(10):1405. Available from: 10.3390/cancers11101405. 10.3390/cancers11101405 PMC6826557 31547070

[65] Dhavale M, Abdelaal MK, Alam ABMN, Blazin T, Mohammed LM, Prajapati D, et al. Androgen Receptor Signaling and the Emergence of Lethal Neuroendocrine Prostate Cancer With the Treatment-Induced Suppression of the Androgen Receptor: A Literature Review. Cureus. 2021;13: e13402. 33754118 10.7759/cureus.13402 PMC7971732

[66] Conteduca V, Ku S-Y, Puca L, Slade M, Fernandez L, Hess J, et al. SLFN11 expression in advanced prostate cancer and response to platinum-based chemotherapy. Mol Cancer Ther. 2020;19:1157–64. 32127465 10.1158/1535-7163.MCT-19-0926 PMC7440143

[67] Zhang B, Stewart CA, Wang Q, Cardnell RJ, Rocha P, Fujimoto J, et al. Dynamic expression of Schlafen 11 (SLFN11) in circulating tumour cells as a liquid biomarker in small cell lung cancer. Br J Cancer. 2022;127:569–76. 35440668 10.1038/s41416-022-01811-9 PMC9346119

[68] Huang J, Cao D, Sha J, Zhu X, Han S. DLL3 is regulated by LIN28B and miR-518d-5p and regulates cell proliferation, migration and chemotherapy response in advanced small cell lung cancer. Biochem Biophys Res Commun. 2019;514:853–60. 31079917 10.1016/j.bbrc.2019.04.130

[69] Ogawa H, Sakai Y, Nishio W, Fujibayashi Y, Nishikubo M, Nishioka Y, et al. DLL3 expression is a predictive marker of sensitivity to adjuvant chemotherapy for pulmonary LCNEC. Thorac Cancer. 2020;11:2561–9. 32691982 10.1111/1759-7714.13574 PMC7471044

[70] Puca L, Gavyert K, Sailer V, Conteduca V, Dardenne E, Sigouros M, et al. Delta-like protein 3 expression and therapeutic targeting in neuroendocrine prostate cancer. Sci Transl Med. 2019;11:eaav0891. 10.1126/scitranslmed.aav0891 PMC6525633 30894499

[71] Obermayr E, Agreiter C, Schuster E, Fabikan H, Weinlinger C, Baluchova K, et al. Molecular Characterization of Circulating Tumor Cells Enriched by A Microfluidic Platform in Patients with Small-Cell Lung Cancer. Cells. 2019;8:880. 31412616 10.3390/cells8080880 PMC6721820

[72] Rudin CM, Reck M, Johnson ML, Blackhall F, Hann CL, Yang JC-H, et al. Emerging therapies targeting the delta-like ligand 3 (DLL3) in small cell lung cancer. Journal of Hematology & Oncology. 2023;16:66. 10.1186/s13045-023-01464-y PMC10290806 37355629

[73] Sardinha M, Palma dos Reis AF, Barreira JV, Fontes Sousa M, Pacey S, Luz R. Antibody-Drug Conjugates in Prostate Cancer: A Systematic Review. Cureus. 2023;15:e34490. 10.7759/cureus.34490 PMC9983052 36874351

[74] Paller CJ, Piana D, Eshleman JR, Riel S, Denmeade SR, Isaacsson Velho P, et al. A pilot study of prostate-specific membrane antigen (PSMA) dynamics in men undergoing treatment for advanced prostate cancer. Prostate. 2019;79:1597–603. 31361358 10.1002/pros.23883 PMC6818502

[75] Sheehan B, Guo C, Neeb A, Paschalis A, Sandhu S, de Bono JS. Prostate-specific Membrane Antigen Biology in Lethal Prostate Cancer and its Therapeutic Implications. Eur Urol Focus. 2022;8:1157–68. 34167925 10.1016/j.euf.2021.06.006

[76] Corpetti M, Müller C, Beltran H, de Bono J, Theurillat J-P. Prostate-Specific Membrane Antigen-Targeted Therapies for Prostate Cancer: Towards Improving Therapeutic Outcomes. Eur Urol. 2024;85:193–204. 38104015 10.1016/j.eururo.2023.11.018

[77] Bakht MK, Derecichei I, Li Y, Ferraiuolo R-M, Dunning M, Oh SW, et al. Neuroendocrine differentiation of prostate cancer leads to PSMA suppression. Endocr Relat Cancer. 2019;26:131–46. 30400059 10.1530/ERC-18-0226

[78] Tsechelidis I, Vrachimis A. PSMA PET in Imaging Prostate Cancer. Front Oncol. 2022;12: 831429. 35155262 10.3389/fonc.2022.831429 PMC8832487

[79] Armstrong AJ, Halabi S, Luo J, Nanus DM, Giannakakou P, Szmulewitz RZ, et al. Prospective Multicenter Validation of Androgen Receptor Splice Variant 7 and Hormone Therapy Resistance in High-Risk Castration-Resistant Prostate Cancer: The PROPHECY Study. JCO. 2019;37:1120–9. 10.1200/JCO.18.01731 PMC6494355 30865549

[80] Løvf M, Zhao S, Axcrona U, Johannessen B, Bakken AC, Carm KT, et al. Multifocal Primary Prostate Cancer Exhibits High Degree of Genomic Heterogeneity. Eur Urol. 2019;75:498–505. 30181068 10.1016/j.eururo.2018.08.009

[81] Scher HI, Graf RP, Schreiber NA, McLaughlin B, Jendrisak A, Wang Y, et al. Phenotypic Heterogeneity of Circulating Tumor Cells Informs Clinical Decisions between AR Signaling Inhibitors and Taxanes in Metastatic Prostate Cancer. Cancer Res. 2017;77:5687–98. 28819021 10.1158/0008-5472.CAN-17-1353 PMC5666339

[82] El-Heliebi A, Hille C, Laxman N, Svedlund J, Haudum C, Ercan E, et al. In Situ Detection and Quantification of AR-V7, AR-FL, PSA, and *KRAS* Point Mutations in Circulating Tumor Cells. Clin Chem. 2018;64:536–46. 29301749 10.1373/clinchem.2017.281295

[83] El-Heliebi A, Kashofer K, Fuchs J, Jahn SW, Viertler C, Matak A, et al. Visualization of tumor heterogeneity by in situ padlock probe technology in colorectal cancer. Histochem Cell Biol. 2017;148:105–15. 28321501 10.1007/s00418-017-1557-5 PMC5508037

[84] Lee AC, Svedlund J, Darai E, Lee Y, Lee D, Lee H-B, et al. OPENchip: an on-chip in situ molecular profiling platform for gene expression analysis and oncogenic mutation detection in single circulating tumour cells. Lab Chip. 2020;20:912–22. 32057051 10.1039/c9lc01248f

[85] Aceto N, Bardia A, Miyamoto DT, Donaldson MC, Wittner BS, Spencer JA, et al. Circulating Tumor Cell Clusters Are Oligoclonal Precursors of Breast Cancer Metastasis. Cell. 2014;158:1110–22. 25171411 10.1016/j.cell.2014.07.013 PMC4149753

[86] Wang C, Zhang Z, Chong W, Luo R, Myers RE, Gu J, et al. Improved Prognostic Stratification Using Circulating Tumor Cell Clusters in Patients with Metastatic Castration-Resistant Prostate Cancer. Cancers (Basel) [Internet]. 2021 [cited 2021 Apr 1];13. Available from: https://www.ncbi.nlm.nih.gov/pmc/articles/PMC7828213/. 10.3390/cancers13020268 PMC7828213 33450815

[87] Lu L, Hu W, Liu B, Yang T. Insights into Circulating Tumor Cell Clusters: A Barometer for Treatment Effects and Prognosis for Prostate Cancer Patients. Cancers. 2022;14:3985. 36010983 10.3390/cancers14163985 PMC9406494

[88] Li D, Wang Y, Li C, Wang Q, Sun B, Zhang H, et al. Cancer-specific calcium nanoregulator suppressing the generation and circulation of circulating tumor cell clusters for enhanced anti-metastasis combinational chemotherapy. Acta Pharm Sin B. 2021;11:3262–71. 34729314 10.1016/j.apsb.2021.04.009 PMC8546850

[89] Kurniali PC, Storandt MH, Jin Z. Utilization of Circulating Tumor Cells in the Management of Solid Tumors. J Pers Med. 2023;13:694. 37109080 10.3390/jpm13040694 PMC10145886

[90] Brady SW, McQuerry JA, Qiao Y, Piccolo SR, Shrestha G, Jenkins DF, et al. Combating subclonal evolution of resistant cancer phenotypes. Nat Commun. 2017;8:1231. 29093439 10.1038/s41467-017-01174-3 PMC5666005

[91] Onozato ML, Yapp C, Richardson D, Sundaresan T, Chahal V, Lee J, et al. Highly Multiplexed Fluorescence in Situ Hybridization for in Situ Genomics. J Mol Diagn. 2019;21:390–407. 30862547 10.1016/j.jmoldx.2019.01.010 PMC6504678

[92] Berg S, Kutra D, Kroeger T, Straehle CN, Kausler BX, Haubold C, et al. ilastik: interactive machine learning for (bio)image analysis. Nat Methods. 2019;16:1226–32. 31570887 10.1038/s41592-019-0582-9

[93] Stringer C, Wang T, Michaelos M, Pachitariu M. Cellpose: a generalist algorithm for cellular segmentation. Nat Methods. 2021;18:100–6. 33318659 10.1038/s41592-020-01018-x

[94] Bouilhol E, Savulescu AF, Lefevre E, Dartigues B, Brackin R, Nikolski M. DeepSpot: A deep neural network for RNA spot enhancement in single-molecule fluorescence in-situ hybridization microscopy images. Biological Imaging. 2022;2: e4. 38510431 10.1017/S2633903X22000034 PMC10951802

[95] Stevens M, Nanou A, Terstappen LWMM, Driemel C, Stoecklein NH, Coumans FAW. StarDist Image Segmentation Improves Circulating Tumor Cell Detection. Cancers (Basel). 2022;14:2916. 35740582 10.3390/cancers14122916 PMC9221404

[96] Wernersson E, Gelali E, Girelli G, Wang S, Castillo D, Langseth CM, et al. Deconwolf enables high-performance deconvolution of widefield fluorescence microscopy image [Internet]. 2022 [cited 2023 Jul 11]. Available from: 10.21203/rs.3.rs-1303463/v1. 10.1038/s41592-024-02294-7 PMC11239506 38844629

[97] Bray M-A, Singh S, Han H, Davis CT, Borgeson B, Hartland C, et al. Cell Painting, a high-content image-based assay for morphological profiling using multiplexed fluorescent dyes. Nat Protoc. 2016;11:1757–74. 27560178 10.1038/nprot.2016.105 PMC5223290

[98] Cimini BA, Chandrasekaran SN, Kost-Alimova M, Miller L, Goodale A, Fritchman B, et al. Optimizing the Cell Painting assay for image-based profiling. Nat Protoc. 2023;18:1981–2013. 37344608 10.1038/s41596-023-00840-9 PMC10536784

[99] Park S, Ang RR, Duffy SP, Bazov J, Chi KN, Black PC, et al. Morphological differences between circulating tumor cells from prostate cancer patients and cultured prostate cancer cells. PLoS ONE. 2014;9: e85264. 24416373 10.1371/journal.pone.0085264 PMC3885705

[100] Zeune LL, Boink YE, van Dalum G, Nanou A, de Wit S, Andree KC, et al. Deep learning of circulating tumour cells. Nat Mach Intell. 2020;2:124–33.

[101] Nanou A, Stoecklein NH, Doerr D, Driemel C, Terstappen LWMM, Coumans FAW. Training an automated circulating tumor cell classifier when the true classification is uncertain. PNAS Nexus. 2024;3:pgae048. 10.1093/pnasnexus/pgae048 PMC10873494 38371418

[102] Bonstingl L, spatialhisto, CTC_CoDuCo [Internet]. Zenodo,. cited 2024 May 7. 2024. Available from: 10.5281/zenodo.11125572.

