# Correction: Advanced single-cell and spatial analysis with high-multiplex characterization of circulating tumor cells and tumor tissue in prostate cancer: unveiling resistance mechanisms with the CoDuCo in situ assay

**DOI:** 10.1186/s40364-024-00698-3

**Published:** 2024-12-02

**Authors:** Lilli Bonstingl, Margret Zinnegger, Katja Sallinger, Karin Pankratz, Christin-Therese Müller, Elisabeth Pritz, Corinna Odar, Christina Skofler, Christine Ulz, Lisa Oberauner-Wappis, Anatol Borrás-Cherrier, Višnja Somođi, Ellen Heitzer, Thomas Kroneis, Thomas Bauernhofer, Amin El-Heliebi

**Affiliations:** 1 Division of Cell Biology, Histology and Embryology, Gottfried Schatz Research Center, Medical University of Graz, 8010 Graz, Austria; 2 Center for Biomarker Research in Medicine (CBmed), 8010 Graz, Austria; 3 European Liquid Biopsy Society (ELBS), 20246 Hamburg, Germany; 4 Diagnostic and Research Center for Molecular BioMedicine, Diagnostic & Research Institute of Pathology, Medical University of Graz, 8010 Graz, Austria; 5 Division of Oncology, Department of Internal Medicine, Medical University of Graz, 8010 Graz, Austria; 6 Diagnostic and Research Center for Molecular BioMedicine, Institute of Human Genetics, Medical University of Graz, 8010 Graz, Austria; 7 Christian Doppler Laboratory for Liquid Biopsies for Early Detection of Cancer, Medical University of Graz, 8010 Graz, Austria; 8 University Comprehensive Cancer Center (CCC) Graz, 8010 Graz, Austria

**Correction: Biomarker Research (2024) 12:140**

10.1186/s40364-024-00680-z

The article originally published with all figures mistakenly absent due to an error by the production team which handled the manuscript. The figures are now included in the final published version.

